# Plasmonic nanostructures through DNA-assisted lithography

**DOI:** 10.1126/sciadv.aap8978

**Published:** 2018-02-02

**Authors:** Boxuan Shen, Veikko Linko, Kosti Tapio, Siim Pikker, Tibebe Lemma, Ashwin Gopinath, Kurt V. Gothelf, Mauri A. Kostiainen, J. Jussi Toppari

**Affiliations:** 1 Department of Physics, Nanoscience Center, P.O. Box 35, 40014 University of Jyväskylä, Finland.; 2 Biohybrid Materials, Department of Bioproducts and Biosystems, Aalto University, 00076 Aalto, Finland.; 3 HYBER Centre of Excellence, Department of Applied Physics, Aalto University, 00076 Aalto, Finland.; 4 Department of Bioengineering, California Institute of Technology, Pasadena, CA 91125, USA.; 5 Centre for DNA Nanotechnology, Interdisciplinary Nanoscience Center, iNANO, Aarhus University, Gustav Wieds Vej 14, 8000 Aarhus C, Denmark.; 6 Department of Chemistry, Aarhus University, Langelandsgade 140, 8000 Aarhus C, Denmark.

## Abstract

Programmable self-assembly of nucleic acids enables the fabrication of custom, precise objects with nanoscale dimensions. These structures can be further harnessed as templates to build novel materials such as metallic nanostructures, which are widely used and explored because of their unique optical properties and their potency to serve as components of novel metamaterials. However, approaches to transfer the spatial information of DNA constructions to metal nanostructures remain a challenge. We report a DNA-assisted lithography (DALI) method that combines the structural versatility of DNA origami with conventional lithography techniques to create discrete, well-defined, and entirely metallic nanostructures with designed plasmonic properties. DALI is a parallel, high-throughput fabrication method compatible with transparent substrates, thus providing an additional advantage for optical measurements, and yields structures with a feature size of ~10 nm. We demonstrate its feasibility by producing metal nanostructures with a chiral plasmonic response and bowtie-shaped nanoantennas for surface-enhanced Raman spectroscopy. We envisage that DALI can be generalized to large substrates, which would subsequently enable scale-up production of diverse metallic nanostructures with tailored plasmonic features.

## Teaser

DALI combines DNA origami with conventional top-down fabrication for creating designer high-resolution plasmonic nanostructures.

## INTRODUCTION

Metallic nanostructures are widely used and explored because of their unique optical properties, such as selective field enhancement via plasmonic resonances, and their potency to serve as components of novel metamaterials (*1*, *2*). However, the currently available fabrication techniques are not feasible for creating complex and sufficiently small metallic shapes for metamaterials functioning at the visible wavelength range. The common wet chemical methods merely yield geometrically limited structures, whereas the standard lithography that allows arbitrary shapes does not provide the required spatial accuracy. Meanwhile, programmable self-assembly of nucleic acids enables the fabrication of custom, precise objects with nanoscale dimensions (*3*, *4*). These structures can be further harnessed as templates for building novel materials and nanodevices with diverse functionalities (*5*–*10*). However, approaches to transfer their spatial information to metal nanostructures have been limited to direct patterning with nanoparticles (*7*, *8*, *11*–*13*) or chemical growth of attached seed particles (*14*–*18*).

Here, we present a parallel, high-throughput DNA-assisted lithography (DALI) method that genuinely combines the high resolution and structural versatility of DNA origami (*19*–*24*) with the robustness of the conventional lithography to create discrete, well-defined, and entirely metallic nanostructures with designed plasmonic properties. DALI is a parallel, high-throughput fabrication method compatible with transparent substrates. The technique facilitates the production of large plasmonic metasurfaces with small (~10 nm) feature sizes, and at present, it is the only viable method for this purpose because of its freedom from costly optical or electron-beam patterning. We demonstrate its feasibility by producing metal nanostructures with chiral plasmonic response and bowtie-shaped nanoantennas for surface-enhanced Raman spectroscopy (SERS). Overall, DALI provides a straightforward approach to overcome the abovementioned prominent obstacles and opens up completely new avenues in nanofabrication.

## RESULTS

**Fig. 1:**
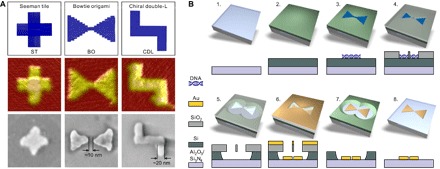
DNA origami designs, a step-by-step fabrication procedure of the DALI method. (**A**) Top: Designed DNA origami shapes (from left to right): ST, BO, and CDL. Middle: AFM images of the folded structures on a mica substrate. Bottom: Scanning electron microscopy (SEM) images of gold nanostructures created by the fabrication method described in detail in (B). The AFM and SEM images are 150 nm × 150 nm in size. (**B**) Steps of the fabrication procedure. Step 1: A transparent sapphire (Al_2_O_3_)/silicon nitride (Si_3_N_4_) chip is freshly cleaned by acetone and isopropanol. Step 2: An amorphous silicon layer is grown on top of the substrate by PECVD. Step 3: The substrate is treated by oxygen plasma, after which the DNA origami nanostructures (BO shown here as an example) are drop-casted on the chip. Step 4: The silicon dioxide (SiO_2_) layer is selectively grown on the bare silicon by CVD, leaving DNA origami–shaped silhouettes in the layer. Step 5: Using the grown SiO_2_ layer as a mask, the silicon underneath is etched away by RIE. Step 6: The metal is deposited onto the chip using PVD in ultrahigh vacuum. Step 7: The SiO_2_ layer is removed in a liftoff process using hydrogen fluoride–based wet etching. Step 8: The remaining silicon is removed by RIE, thus leaving the DNA origami–shaped metal nanostructure on the substrate.

We prepared four different DNA origami designs: the so-called Seeman tile (ST) (*25*), a bowtie origami (BO), and two versions of a chiral double-L (CDL), that is, CDL with and without protruding staple strand extensions to control its landing orientation on a silicon substrate (Fig. 1A, top). The origami structures were folded in a thermal annealing process with high yield, and the structural integrity of each design was verified by atomic force microscopy (AFM) (Fig. 1A, middle) and agarose gel electrophoresis (see fig. S1). To demonstrate the versatility of DALI (Fig. 1B), we converted the abovementioned DNA designs to discrete one-to-one metallic shapes (Fig. 1A, bottom) and characterized their plasmonic properties in detail.

The folded origami structures were deposited without any purification onto an oxygen plasma–cleaned substrate consisting of a transparent bottom layer [sapphire (Al_2_O_3_) or silicon nitride (Si_3_N_4_)] and a top layer of amorphous silicon, grown by plasma-enhanced chemical vapor deposition (PECVD) (Fig. 1B, steps 1 to 3). After the deposition, a silicon dioxide (SiO_2_) layer was formed on top of the silicon in a selective CVD process (Fig. 1B, step 4) (*26*, *27*). The oxide primarily grows on the bare areas of the silicon and only slightly on top of the deposited DNA origami. The thin oxide layer on top of the origami can be easily removed by short reactive ion etching (RIE). Therefore, the formed oxide layer can be further used as a stencil for the following process steps.

**Fig. 2:**
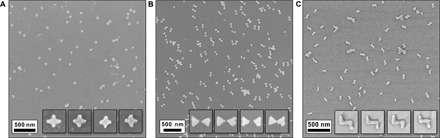
Large-area SEM images of the structures created with DALI. (**A**) AuPd ST structures on Si_3_N_4_. (**B**) Au BO structures on Si_3_N_4_. (**C**) Au CDL structures on sapphire. All the inset images are 150 nm × 150 nm in size.

The rest of the procedure starts with isotropic RIE etching of the silicon underneath the oxide film through the origami-shaped holes (Fig. 1B, step 5). This step is followed by a physical vapor deposition (PVD) of a gold film under ultrahigh vacuum conditions (Fig. 1B, step 6). Finally, the silicon dioxide stencil and the remaining silicon are etched away (Fig. 1B, steps 7 and 8), which results in discrete origami-shaped metal nanostructures on the transparent substrate (Fig. 2). The produced metal structures are uniform in size and shape, and the density of the structures can be easily tuned by varying the parameters of the deposition step (step 3). Most of all, the parallel fabrication provides high throughput and resolution with a feature size of ~10 nm; the width of the arms for STs and CDLs is ~20 nm, and the gap size for the BOs can be less than 10 nm (Figs. 1A and 2B). The detailed fabrication procedure is presented in note S1.

Besides its capability of fabricating nanostructures with specific shapes (as ST and CDL) and with relatively high yields (see the “Fabrication yield analysis” section in the Appendix of the Supplementary Materials), DALI can also be used to produce particle pairs that are separated by adjustable nanoscale gaps. An example of this structure is a bowtie-shaped optical antenna, the shape that has been widely harnessed in different optical applications. This is due to its characteristic highly confined plasmonic mode within the gap that provides an extreme field enhancement. For applications such as surface-enhanced sensing (*28*) and fluorescence enhancement (*29*), it is essential to tune the wavelength of the maximum enhancement, which substantially depends on the selected material, size, and exact shape of the structure, in particular on the sharpness of the tips and the size of the gap. To obtain the maximum enhancement within the visible wavelength range, the size of the gap between the antennas needs to be below 10 nm on a glass substrate (*29*, *30*). To date, this resolution has only been achieved by focused beam lithography (electron or ion beam). This is an expensive scanning technique, which makes it unsuitable for large-scale production.

To demonstrate that our DALI method provides the necessary accuracy to produce efficient optical bowtie antennas, we used the designed BO shape (Fig. 1A) as a template. The gap between the two triangles can be formed because the thin silicon oxide layer grown on top of the origami (fabrication step 4) appears to be thicker at the narrow part of the BO than at the triangles. The reason is that the silicon oxide grows slowly on top of the BO starting from the edges of the already formed oxide layer, and these edges can merge at the thin bridge between the two triangles, resulting in a nonuniform thickness profile of the oxide. Using careful etching techniques in step 5, we obtain a stencil mask with two individual triangles separated by a nanometer-scale gap. By merely controlling the processing times in fabrication steps 4 and 5, the gap size and therefore the resulting plasmonic resonance can be tuned (see note S2). This feature enabled us to fabricate the smallest, entirely metallic bowtie antenna that has ever been reported (~125 nm in the long axis including two sub–60-nm triangles and a gap ranging from 5 to 20 nm), verified by detailed characterization of the optical/plasmonic properties of the produced structures (see below). In addition, to show the uniformity of DALI-fabricated structures, we analyzed numerous bowtie antennas from one fabrication batch and determined the average gap size, thickness, and bending angle, that is, angle between the triangles. We obtained 12 ± 5 nm (SD) for the gap size, 21.6 ± 0.2 nm (SD) with ~2-nm mean roughness for the thickness, and 179 ± 12° (SD) for the bending angle (note S1).

For plasmonic characterization, we measured the scattering spectra at different polarization angles from single metallic nanoantennas (note S3) using a dark-field microscope coupled to a spectrometer via an optical fiber (details in note S4). This allowed us to separately determine the localized surface plasmon resonances (LSPRs) of the structure both along and perpendicular to the gap. As a control and as a comparison, we also characterized the metallic ST shapes.

**Fig. 3:**
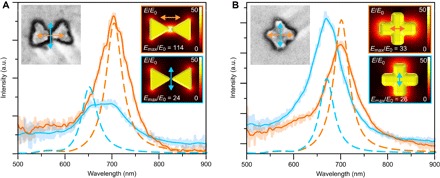
Experimental and simulated linear polarization scattering spectra for single BO- and ST-shaped gold nanostructures. (**A** and **B**) Dark-field scattering spectra of the BO and ST shapes (SEM images shown in the left insets, 200 nm × 200 nm in size) measured at two different polarization angles (indicated by correspondingly colored arrows in the insets). Lines show the smoothed spectra, whereas the original data are shown faded in the background. Dashed lines show simulated scattering spectra for the BO and ST shapes in (A) and (B), respectively. The right-side insets show a field enhancement, that is, the local electric field (*E*) divided by the incoming electric field (*E*_0_) at the chosen polarization angles (shown by colored arrows) at the middle height of the structure, that is, 10 nm above the surface. The main LSPR modes are at 650 and 705 nm for the BO and at 670 and 700 nm for the slightly asymmetric ST. The maximum local field enhancement, *E*_max_/*E*_0_, for each sample is also stated in each inset. a.u., arbitrary units.

For the BO shapes, we observed the highest LSPR peaks at the polarizations along the gap (λ_max_ at 704 nm) and perpendicular to the gap (λ_max_ at 650 to 700 nm), as shown in Fig. 3A (additional spectra from different samples shown in note S5). The LSPR peak associated with the gap mode has higher intensity and longer wavelength than the perpendicular mode, which is in good agreement with the earlier reports (*28*–*30*) and with the finite element method (FEM) simulated data shown in Fig. 3A. The simulated spatial distributions of the field enhancement for these two main modes are plotted in the insets of Fig. 3A. Because our structure is smaller than the previously reported bowties, the observed LSPR peaks accordingly appear at shorter wavelengths (despite the fact that the sapphire substrate has a higher refractive index than indium tin oxide/glass substrates used in the earlier studies) (*29*, *30*).

To estimate the LSPR variations within a single fabrication batch, we simulated the structures with geometries differing from the optimal shape by the amount of the observed SD (see above). The bending angle change within the SD had no noticeable effect on the LSPR peak position or the field enhancement. However, an increase in the thickness (by mean roughness) or in the gap size (by SD) yielded 8.5- or 12.5-nm blue shifts in the gap mode resonance, respectively. Moreover, these changes induced 12 and 30% decreases in the field enhancement. Only the thickness variation had an effect on the perpendicular mode, inducing a 6.5-nm blue shift with a negligible decrease in the field enhancement. In summary, the changes in plasmonic properties due to the observed variations in the fabrication process were insignificant, thus revealing the homogeneity of the DALI process (for more details, see fig. S15).

In the case of metallic ST shapes, we typically observed the highest LSPR peaks at polarizations along the arms of the cross (Fig. 3B). For a fully symmetric ST, these peaks should overlap, but due to an unavoidable slight asymmetry between the two arms, the peaks usually appeared at slightly different wavelengths. This is in qualitative agreement with the simulated data shown in Fig. 3B. The LSPR resonances for the BO and ST structures lay within the same wavelength region, but the key difference is the obtained maximum field enhancement. As can be seen from the simulation results in the insets of Fig. 3 (A and B), the excitation of the BO gap mode should yield a maximum field enhancement almost four times as high as that of the other modes, which all have very similar responses.

**Fig. 4:**
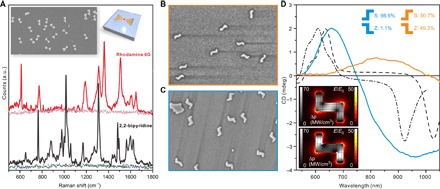
Metallic BO shapes for SERS and CDL shapes for creating chiral plasmonic response. (**A**) Raman spectra (baseline-corrected) measured from a sample containing nanosized gold BOs coated with either the rhodamine 6G (red) or 2,2-bipyridine molecules (black). Lighter dotted lines show the response of the same concentration of the molecules on a pure substrate without the BOs. (**B**) SEM of a sample containing 50:50 distribution of S- and Z-shaped metallic CDLs on a sapphire substrate. (**C**) SEM of a sample with ~99% of the CDLs in the S-configuration on a sapphire substrate. The SEM images (B and C) are 1.4 μm × 1 μm in size. (**D**) Orange and blue curves: Averaged and baseline-corrected CD spectra measured from the samples shown in (B) and (C). Black lines: Simulated CD response for a symmetric (dashed) and slightly asymmetric (dash-dotted) S-shaped CDL. Insets show the field enhancement (color scale) and power loss inside the metal (grayscale) for the symmetric CDL and for the right-handed (upper) and left-handed (lower) circularly polarized 640-nm excitation light.

The abovementioned LSPR measurement can be used to verify the desired plasmonic activity of the fabricated nanostructures. Yet, for more application-oriented purposes and to verify the effective plasmonic hotspots, we demonstrated the feasibility of the BO antennas for SERS by using two typical SERS markers with opposite charges (namely, rhodamine 6G and 2,2-bipyridine). The samples were fabricated by pipetting a droplet of the chosen 1 μM marker solution onto a sapphire chip so that the droplet covered both the prefabricated metallic BOs and the clean sapphire surface (clean surface was used as a reference). After drying the droplet, we characterized the chip using Raman microscopy. The characteristic Raman spectra of both markers were detected with significantly high resolution (Fig. 4A) on the areas covered by the BO antennas (Fig. 4A, inset), whereas no Raman signal was observed from the empty areas of the chip (Fig. 4A, dotted lines).

As a final demonstration of the versatility of the DALI method, we fabricated planar chiral structures, which are of fundamental importance in metamaterials and in biosensing applications, especially in enhancing the detection of different molecular enantiomers. In particular, the miniaturization of these structures to the sub–100-nm scale is essential to fabricate metamaterials that function in the visible spectrum. To produce a controlled chiral optical response within this wavelength range, we designed a CDL-shaped origami with suitable dimensions. However, to produce a sample with the desired response, most of the CDL origamis should have a specified orientation after the deposition, that is, CDLs should land on the O_2_ plasma–treated Si substrate in either S- or Z-shaped orientation. We observed that when 37.5 mM Mg^2+^ was used in the deposition buffer, ~80% of the plain CDL origamis tend to adsorb onto the Si surface with the S-shaped orientation. However, at 70 mM or higher Mg^2+^ concentration, the deposition yielded equal amounts of S- and Z-shaped orientations. Besides merely adjusting the Mg^2+^ concentration, it is possible to efficiently alter the distribution of S- and Z-shaped orientations by incorporating dozens of protruding staple strand extensions into the design. The reason is that if these single-stranded DNA (ssDNA) protrusions are attached only to the one side of the origami, then the strands act as an entropic brush, and therefore, the structures prefer to land with the staple strand extension side up. Here, by introducing a total of 114 poly-T_20_ overhangs into the design, the bias toward the S-shaped orientation could be increased up to 99% (see the “Fabrication yield analysis” section in the Appendix of the Supplementary Materials).

By converting the CDL origami into metallic nanostructures, we could produce planar chiral samples with diverse CD responses (Fig. 4D) depending on the deposition process. A sample with randomly oriented CDLs (equal amount of S- and Z-shaped orientations, unmodified CDL) (Fig. 4B) and a sample with S-shaped orientation–biased CDLs (“swimmer strand”–equipped CDL) (Fig. 4C) exhibited very different broadband CD effects in the wavelength range of 500 to 1050 nm. The sapphire chip with only S-shaped gold nanostructures showed a well-defined peak at 650 nm and a wide negative peak at 950 nm, which qualitatively agrees with the FEM simulations (black lines in Fig. 4D). The simulated power loss inside the metal shows a clear difference between the two opposite circularly polarized excitations (Fig. 4D, insets), whereas the effect in the local field enhancement is small, which is also clear from the matching absorption spectra between the S- and Z-shaped orientations (note S6). This confirms that the observed CD response is due to the chiral shape of the metal structures.

The broadness of the negative peak at 950 nm compared to the simulations can be attributed to a variation of the length in the end pieces of the fabricated metallic CDLs, which is also visible in Fig. 4 (B and C). The two simulations show a CD response of a fully symmetric CDL structure along the origami dimensions (dashed line) and a similar asymmetric structure with the end pieces asymmetrically shortened (dash-dotted line). This shows that the peak at 640 nm is almost unaffected, whereas the negative peak shifts considerably, thus providing a wide feature when averaged over the distribution of symmetric and asymmetric CDLs. The CD spectrum of the randomly oriented sample showed only insignificant broad spectral features, which demonstrate the lack of preferred surface orientation. It is noteworthy that despite a very small optical density of the setup (perpendicular incident light passing through a monolayer of 20-nm-thick CDLs), the CD effect was clearly observed. This strongly implies that the sensitivity could be further improved, thus opening up potential applications in chiral molecule sensing.

## DISCUSSION

The DALI method enables highly parallel fabrication of arbitrary-shaped plasmonic nanostructures with well-defined shapes using self-assembled DNA origami templates. Because DNA origami designs are straightforward to produce in high yields and, in DALI, do not require any additional treatment or purification steps, the method is robust and provides high throughput. We demonstrated this by fabricating three different metallic nanoshapes on transparent substrates, including bowtie nanoantennas with controllable plasmonic resonances at the visible range (the minimum bowtie gap that was characterized was 8 nm) and planar CDL structures that provide a clear CD response. As a final note, we believe that the cheap wafer-scale production of plasmonic metasurfaces can be achieved using the DALI method because it does not rely on costly patterning methods and is compatible with various deposition techniques (*10*, *31*, *32*) for large lattice structures spatially ordered in both position and orientation. Furthermore, we are positive that this novel method can find intriguing applications in biosensing and in the fabrication of bioinspired surfaces and dielectric metamaterials (*33*).

## MATERIALS AND METHODS

### Design of DNA structures

The BO and CDL were designed using caDNAno (*34*) and CanDo (*35*, *36*) software. ST was fabricated as explained by Liu *et al*. (*25*). All the structures are based on the 7249-nucleotide-long M13mp18 plasmid scaffold strand. The BO structure is twist-corrected, that is, the twist resulting from the square lattice packing (*35*–*37*) is relaxed by additional base skips in the design (*35*, *36*, *38*) according to a CanDo simulation. Side strands in the BO and CDL designs contain TTTTTTTT (poly-T_8_) overhangs to prevent the blunt-end stacking of the origami structures. In the case of ST, the side strands were omitted (*25*). Two different versions of the CDL were fabricated: a plain unmodified version and a version with the staple strand extensions at one side. These extensions were used to bias the landing orientation of the CDL structure (Fig. 4, B and C). The folding and characterization procedures for all the origami shapes are described below. The caDNAno designs for the BO and CDL and the complete list of staple strands are presented in the Appendix of the Supplementary Materials. The staple extensions (114 strands) of the CDL replace one to one the strands from the set of “core strands pool #1” (green color in the caDNAno design, see the Appendix in the Supplementary Materials). These strands hybridize to the scaffold via the same sequence as core strands pool #1, but the extended strands contain poly-T_20_ overhangs at the 5′-end. The protrusions are designed in such a way that the CDL will adopt an S-shaped orientation when deposited onto the substrate, that is, all the poly-T extensions are pointing out from the substrate and the unmodified side of the CDL is attached to the substrate.

### DNA origami fabrication

The DNA origami structures were prepared as 100-μl quantities by mixing the following components: (i) 40 μl of 2.5× TAE buffer with Mg^2+^ [100 mM tris, 47.5 mM acetic acid, 2.5 mM EDTA, 31.25 mM MgCl_2_ (pH ~8.3)]; (ii) 40 μl of staple strand mix (each staple at ~500 nM concentration) [The staple strand mix was prepared by pipetting equal amounts of each staple strand (Integrated DNA Technologies, initial concentration of 100 μM in water, ~200 strands per design) and mixing them together. This resulted in about 500 nM concentration for each strand in the staple strand mix. If the origami contains less than 200 strands (as in ST: 177 strands), then 500 nM concentration was achieved by dilution with water]; and (iii) 20 μl of the scaffold strand M13mp18 (100 nM in water) (Tilibit Nanosystems).

The final concentration of the scaffold strand in a total reaction volume of 100 μl was 20 nM, and the concentration of magnesium was 12.5 mM. The staple strands were used in 10× excess compared to the M13mp18 scaffold strand (each staple at 200 nM concentration). The following thermal annealing ramp was used for folding: From 90° to 70°C with increments of 0.2°C/8 s, from 70° to 60°C with increments of 0.1°C/8 s, and from 60° to 27°C with increments of 0.1°C/2 min. Finally, the solution was stored at 12°C. After folding, the theoretical maximum concentration of DNA origami is 20 nM.

### Gel electrophoresis for DNA origami

Gel electrophoresis was used to verify the quality of the DNA origami folding (see fig. S1). Agarose gels (2%) were prepared by dissolving 2 g of agarose (Bioline, Molecular Grade) into 100 ml of 1× TAE buffer with 11 mM Mg^2+^ (40 mM tris, 19 mM acetic acid, 1 mM EDTA, 11 mM MgCl_2_). The gel was stained using 100 μl of ethidium bromide solution (0.625 mg/ml). Ten microliters of DNA origami solution were mixed with 2 μl of 6× loading dye (New England Biolabs). M13mp18 ssDNA (at 100 nM) was used as a reference at 40 nM concentration by diluting it with the folding buffer (1× TAE buffer with 12.5 mM Mg^2+^). The gel wells were loaded with 10 μl of the sample. The gels were run with a constant voltage of 90 V for 45 to 60 min and imaged under ultraviolet (UV) light (Bio-Rad equipment, Image Lab Software).

### Atomic force microscopy

The AFM (Veeco, Dimension 3100) was used to characterize the sample after the DNA origami deposition and to verify the successful formation of the origami-shaped opening in the SiO_2_. The AFM imaging of these openings was usually carried out in tapping mode with a scan size varying from 1 to 10 μm. The tip velocity ranged from 2 to 15 μm/s. For the analysis of the uniformity of the DALI-fabricated metallic nanostructure, numerous bowtie antennas from a single batch were characterized using the peak force tapping mode in air (Bruker, Icon), where the scan size was from 1 to 5 μm and the tip velocity was from 1.5 to 10 μm/s.

### Scanning electron microscopy

Sapphire is an insulator, and thus, imaging with a scanning electron microscope (Raith eLine scanning electron microscopy system) induces charging of the substrate. To reduce the distortion caused by the charging effect, low acceleration voltages (5 to 10 kV) were used. In addition, the scanning times were minimized to reduce the current exposed to a small area. The images were taken with a 30-μm aperture in a continuous averaging mode. Brightness and contrast levels were adjusted to compensate for the charging effect.

### DNA-assisted lithography

See note S1 for details about general lithography materials and methods. Details about sample preparation for single-particle LSPR measurement can be found in note S3.

### Single-particle linear polarization LSPR measurement

To measure the single-particle spectra, an Olympus BX51TRF microscope with Olympus MPLANFL N objectives (5×/20×/50×) was used for imaging. The excitation light was generated using a halogen lamp (Olympus 100 W) inside a lamp housing (Olympus U-LH100IR-1-7). Dark-field imaging was carried out using a dark-field condenser (Olympus U-DCW). An analyzer (Olympus U-AN360-3) was used to select the polarization of the measured light, and a linear polarizer (Thorlabs LPVISE200-A 2”) was used to align the analyzer. A custom-made slit mask was used to align the polarizer and the marker grid of the sample. The light was extracted by placing a fiber (Thorlabs UM22-300-custom; core size, 300 μm) onto the output port of the microscope and by focusing the tip of the fiber to the image plane of the port. The other end of the fiber was connected to a collimator (Thorlabs) that was, in turn, connected to a custom-made switch box (Thorlabs). The measured light was focused to the slit of the spectrograph [Princeton Instruments SP2150 (Acton)] equipped with a charge-coupled device (CCD) camera (Andor iVac DR-324B-FI) to record the spectra. The dark-field images were recorded using a Canon EO5 6D camera connected to the other microscope port. The whole setup was located inside an electromagnetically shielded room, and the spectrograph and the CCD camera were connected to a computer using a Black Box IC404A fiber extender. A Thorlabs M530L3-mounted light-emitting diode connected to the switch box was used to visualize the position of the fiber spot on the sample, whereas a *XYZ*-micrometer translational stage was used to move the fiber in the microscope view and to focus the fiber spot (fig. S10A). Andor Solis (version 4.18) was used to measure the spectra, and Canon EOS utility was used to take the dark-field images remotely. The single-particle spectroscopy (SPS) microscope setup is schematically illustrated in fig. S10C (see note S4 for details).

### CD measurement of CDL samples

The CD measurements of the CDLs on a sapphire substrate were carried out using a Jasco J-715 CD spectrometer. The sample surface was placed perpendicularly to the incident beam. For all the samples measured, the illumination direction was from the back side (sapphire side) to the particle side. A measurement with a reverse direction would yield the same result due to the symmetry. In addition, a square aperture of 4 mm × 4 mm was used in direct contact to the back side of the sample to reduce the diffraction of the sample edges.

### Surface-enhanced Raman spectroscopy

The SERS measurements were carried out using a Senterra dispersive Raman microscope (BRUKER). Confocal Raman spectroscopy was used to acquire SERS of the molecule-BO samples. Raman scattering was detected using a Peltier-cooled (−70°C) CCD camera (255 × 1024 pixels), focusing only within the fingerprint regions (500 to 1800 cm^−1^). Detection was carried out using 180° geometry and a near-infrared diode laser (785 nm) for the excitation. The spectrometer was equipped with a diffraction grating (1200 grooves/mm), and the slit provided a spectral resolution of 2 cm^−1^. The laser power at the sample ranged from 1 to 10 mW, and the acquisition time ranged from 1 to 5 s. The area of the laser spot on the samples was approximately 1 μm in diameter. The molecular markers with 1 μM concentration in ethanol were drop-casted on the BO sample surface before the measurement. The setup was calibrated using built-in templates and internal Raman standards.

## Supplementary Material

http://advances.sciencemag.org/cgi/content/full/4/2/eaap8978/DC1

## SUPPLEMENTARY MATERIALS

Supplementary material for this article is available at http://advances.sciencemag.org/cgi/content/full/4/2/eaap8978/DC1 note S1. DALI. note S2. Gap formation in a BO structure. note S3. Single-particle LSPR sample fabrication. note S4. Single-particle linear polarization LSPR measurement. note S5. Additional single-particle linear polarization LSPR spectra. note S6. UV-Vis measurement of CDL samples. note S7. Numerical simulations. fig. S1. Agarose gel electrophoresis of DNA origamis. fig. S2. DNA origami deposition on the Si surface. fig. S3. Schematic view of the reaction chamber setup for the SiO_2_ growth. fig. S4. Fabrication of trenches/silhouettes with different DNA origami shapes. fig. S5. Isotropic RIE etching of silicon. fig. S6. PVD of gold. fig. S7. HF liftoff (removal of the SiO_2_ mask). fig. S8. AFM images with the corresponding thickness profiles and a SEM image of Au bowtie antennas on a sapphire substrate. fig. S9. Schematic illustration of the oxide growth in the vicinity of the BO on a Si substrate. fig. S10. Schematics of the SPS setup. fig. S11. Single-structure spectra of different metallized origami shapes. fig. S12. Normalized UV-Vis spectra of CDL samples with S-configuration and random orientation. fig. S13. Simulation geometry for a CDL particle (S-shaped orientation) with a clockwise polarized incident light and used mesh. fig. S14. Geometries of the different types of particles for the Comsol simulations. fig. S15. Simulated LSPR spectra and field enhancements (*E*/*E*_0_ at resonance frequency) for the optimal bowtie structure and for the structures with geometries altered by the amount of the observed SDs. table S1. Parameters for a-Si CVD. table S2. Parameters for O_2_ plasma RIE. table S3. Parameters for RIE SiO_2_ etching. table S4. Parameters for RIE Si etching. Appendix Design and sequences of BO Design and sequences of CDL Additional SEM data set Fabrication yield analysis References (*39*–*41*)
