# Supplementary material for: Plasmonic nanostructures through DNA-assisted lithography
Source: Sci Adv. 2018 Feb 2;4(2):eaap8978. doi: 10.1126/sciadv.aap8978 (PMC5804581; doi:10.1126/sciadv.aap8978)
Supplement: http://advances.sciencemag.org/cgi/content/full/4/2/eaap8978/DC1 [file supp_4_2_eaap8978__index.html]

Science Advances | Science Advances

## Supplementary Materials

**This PDF file includes:**

- note S1. DALI.
- note S2. Gap formation in a BO structure.
- note S3. Single-particle LSPR sample fabrication.
- note S4. Single-particle linear polarization LSPR measurement.
- note S5. Additional single-particle linear polarization LSPR spectra.
- note S6. UV-Vis measurement of CDL samples.
- note S7. Numerical simulations.
- fig. S1. Agarose gel electrophoresis of DNA origamis.
- fig. S2. DNA origami deposition on the Si surface.
- fig. S3. Schematic view of the reaction chamber setup for the SiO2 growth.
- fig. S4. Fabrication of trenches/silhouettes with different DNA origami shapes.
- fig. S5. Isotropic RIE etching of silicon.
- fig. S6. PVD of gold.
- fig. S7. HF liftoff (removal of the SiO2 mask).
- fig. S8. AFM images with the corresponding thickness profiles and a SEM image of Au bowtie antennas on a sapphire substrate.
- fig. S9. Schematic illustration of the oxide growth in the vicinity of the BO on a Si substrate.
- fig. S10. Schematics of the SPS setup.
- fig. S11. Single-structure spectra of different metallized origami shapes.
- fig. S12. Normalized UV-Vis spectra of CDL samples with S-configuration and random orientation.
- fig. S13. Simulation geometry for a CDL particle (S-shaped orientation) with a clockwise polarized incident light and used mesh.
- fig. S14. Geometries of the different types of particles for the Comsol simulations.
- fig. S15. Simulated LSPR spectra and field enhancements (*E/E*0 at resonance frequency) for the optimal bowtie structure and for the structures with geometries altered by the amount of the observed SDs.
- table S1. Parameters for a-Si CVD.
- table S2. Parameters for O2 plasma RIE.
- table S3. Parameters for RIE SiO2 etching.
- table S4. Parameters for RIE Si etching.
- Appendix
- Design and sequences of BO
- Design and sequences of CDL
- Additional SEM data set
- Fabrication yield analysis
- References (*39–41*)

Download PDF

**Files in this Data Supplement:**

- Adobe PDF - aap8978\_SM.pdf
