# Supplementary material for: Plasmonic nanostructures through DNA-assisted lithography
Source: Sci Adv. 2018 Feb 2;4(2):eaap8978. doi: 10.1126/sciadv.aap8978 (PMC5804581; doi:10.1126/sciadv.aap8978)
Supplement: http://advances.sciencemag.org/cgi/content/full/4/2/eaap8978/DC1 [file aap8978_SM.pdf]

Science Advances

AAAS

advances.sciencemag.org/cgi/content/full/4/2/eaap8978/DC1

Supplementary Materials for

Plasmonic nanostructures through DNA-assisted lithography

Boxuan Shen, Veikko Linko, Kosti Tapio, Siim Pikker, Tibebe Lemma, Ashwin Gopinath, Kurt V. Gothelf, Mauri A. Kostiainen, J. Jussi Toppari

Published 2 February 2018, *Sci. Adv.* **4**, eaap8978 (2018)
DOI: 10.1126/sciadv.aap8978

This PDF file includes:

- note S1. DALI.
- note S2. Gap formation in a BO structure.
- note S3. Single-particle LSPR sample fabrication.
- note S4. Single-particle linear polarization LSPR measurement.
- note S5. Additional single-particle linear polarization LSPR spectra.
- note S6. UV-Vis measurement of CDL samples.
- note S7. Numerical simulations.
- fig. S1. Agarose gel electrophoresis of DNA origamis.
- fig. S2. DNA origami deposition on the Si surface.
- fig. S3. Schematic view of the reaction chamber setup for the SiO_2 growth.
- fig. S4. Fabrication of trenches/silhouettes with different DNA origami shapes.
- fig. S5. Isotropic RIE etching of silicon.
- fig. S6. PVD of gold.
- fig. S7. HF liftoff (removal of the SiO_2 mask).
- fig. S8. AFM images with the corresponding thickness profiles and a SEM image of Au bowtie antennas on a sapphire substrate.
- fig. S9. Schematic illustration of the oxide growth in the vicinity of the BO on a Si substrate.
- fig. S10. Schematics of the SPS setup.
- fig. S11. Single-structure spectra of different metallized origami shapes.
- fig. S12. Normalized UV-Vis spectra of CDL samples with S-configuration and random orientation.
- fig. S13. Simulation geometry for a CDL particle (S-shaped orientation) with a clockwise polarized incident light and used mesh.
- fig. S14. Geometries of the different types of particles for the Comsol simulations.

- fig. S15. Simulated LSPR spectra and field enhancements (E/E_0 at resonance frequency) for the optimal bowtie structure and for the structures with geometries altered by the amount of the observed SDs.
- table S1. Parameters for a-Si CVD.
- table S2. Parameters for O_2 plasma RIE.
- table S3. Parameters for RIE SiO_2 etching.
- table S4. Parameters for RIE Si etching.
- Appendix
- Design and sequences of BO
- Design and sequences of CDL
- Additional SEM data set
- Fabrication yield analysis
- References (*39*–*41*)

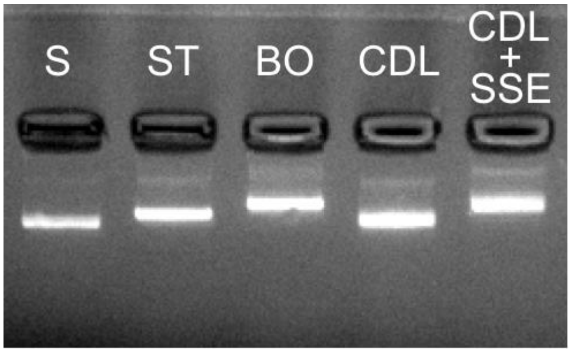

fig. S1. Agarose gel electrophoresis of DNA origamis. S = scaffold strand (M13mp18), ST = Seeman tile, BO = bowtie origami, CDL = chiral double-L, CDL+SSE = CDL equipped with staple strand extensions (SSEs).

note S1. DALI.

1) Substrate preparation

Silicon nitride (Si_3N_4) or sapphire (Al_2O_3) wafer was first cut into 7 mm × 7 mm chips, which were then cleaned in hot acetone and sonicated in isopropanol for 2 minutes. A cotton stick was used to gently remove the glue residues from the chips after cutting the pieces with a silicon saw. The substrate was dried by nitrogen flow after cleaning.

2) Chemical vapor deposition (CVD) of a silicon layer on top of the substrate

The amorphous silicon (a-Si) layer on the substrate was deposited by using the Plasmalab 80+ PECVD equipment (Oxford instruments). The process parameters are listed in table S1. The deposition rate is roughly 36 nm/min. The deposition time of 1.5 min will thus result in an about 54-nm thick a-Si layer.

table S1. Parameters for a-Si CVD.

5 % SiH_4 in 95 % N_2 flow	500 sccm
Pressure	1000 mTorr
RF power	15 W
Temperature	250 °C
Time	1.5 min

3) DNA origami deposition onto the substrate

The Si layer was first treated by O_2 plasma to make it hydrophilic and negatively charged. After this treatment, the DNA origami can efficiently adsorb onto the substrate with the help of Mg^{2+} ions. In addition, it has been noticed that the roughness of the CVD deposited Si layer can be reduced if a prolonged O_2 plasma treatment with high power is used. The O_2 plasma etching was carried out using the Plasmalab 80+ RIE equipment (Oxford instruments). The process parameters are listed in table S2. After the O_2 plasma treatment, the substrate was typically used within the next 30 min.

table S2. Parameters for O_2 plasma RIE.

O_2	50 sccm
Pressure	40 mTorr
RF power	200 W
Temperature	30 °C
Time	20 min

For the deposition, the DNA origami solution (20 nM concentration) was mixed with 1× TAE buffer with 500 mM Mg^{2+} in order to increase Mg^{2+} concentration and therefore enhance the adsorption. Different Mg^{2+} concentrations were tested, but for the general purpose, 100 mM final Mg^{2+} concentration was used (lower than 100 mM Mg^{2+} concentration was used only for CDL origami for selective deposition). Typically, 10 µl of ~10 nM DNA origami solution with elevated Mg^{2+} concentration was drop-casted on a substrate and incubated for 5 min. Then the sample surface was gently washed with 60 µl of ddH_2O for 3 to 4 times and dried by N_2 flow. A typical sample of a ST deposited on a Si surface is shown in fig. S2.

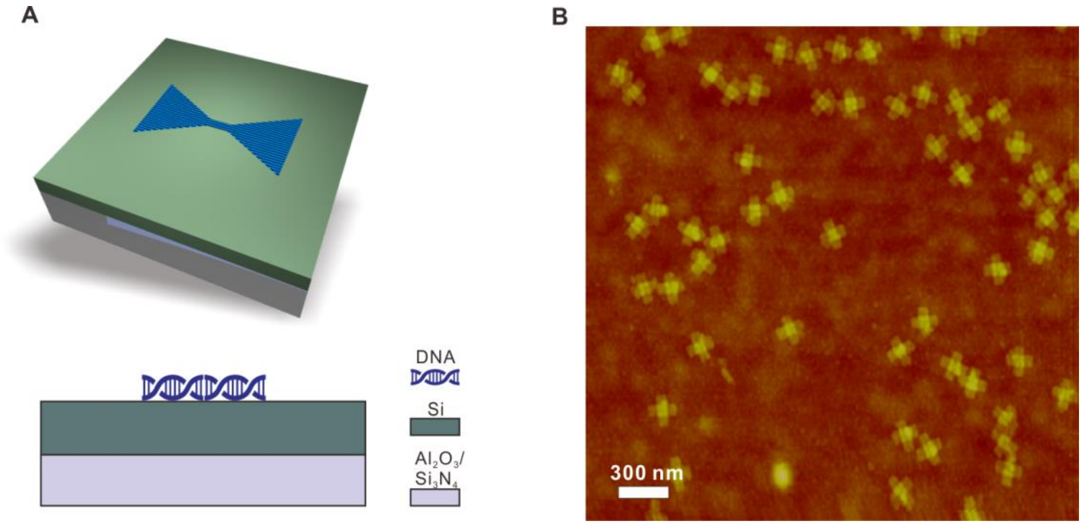

fig. S2. DNA origami deposition on the Si surface. (**A**), Schematic view of the deposition step with the BO-shape. (**B**), AFM micrograph of STs immobilized on a Si surface.

4) Negative SiO_2 mask growth

To selectively grow a SiO_2 layer with DNA origami silhouettes (serves as a hard mask for metal evaporation) a room temperature CVD protocol from reference (*26*) was used and slightly modified. Two small vials containing 10 mL of tetraethyl orthosilicate (TEOS) and 10 mL of 25 % ammonium hydroxide (NH_4OH) were placed into a 1.5 L desiccator. To improve the reproducibility, a silica gel cured in a climatic test chamber (WK3-180/40 R300, Weiss Umwelttechnik) was employed as shown in fig. S3. The SiO_2 growth rate can be adjusted by changing the curing conditions of the silica gel. For a typical process, 100 g of silica gel was cured at room temperature, 80 % relative humidity (RH) for 20 hours. In our case, the cured silica gel can absorb roughly 30 g of water. Incubation in lower humidity and a shorter incubation time decrease the deposition rate but yield a smoother film.

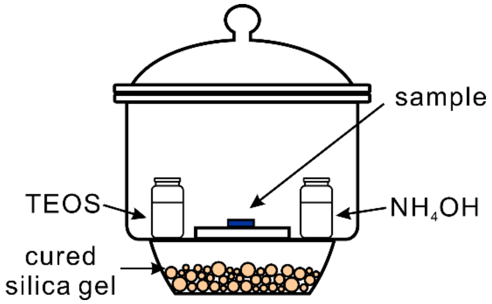

fig. S3. Schematic view of the reaction chamber setup for the SiO_2 growth. TEOS and NH_4OH are placed beside the sample containing the deposited DNA origamis.

Here, we used 16–22 hour SiO_2 growth time. The growth time was varied mainly for the following reasons. First, the SiO_2 growth is not only in perpendicular direction to the sample surface. As the film grows thicker, the opening of the DNA origami silhouette becomes smaller. By utilizing this feature, *i.e*. by varying the growth time, the gap size of the BO could also be tuned (more details in Note S7). The other factor that needs to be taken into account is the aging of TEOS. It was observed that the growth rate of SiO_2 layer was decreased after a bottle of TEOS was opened for a few months. Therefore, the reaction time needs to be slightly adjusted to get the desired results. AFM images of the samples with different origami shapes after the SiO_2 layer deposition are shown in fig. S4.

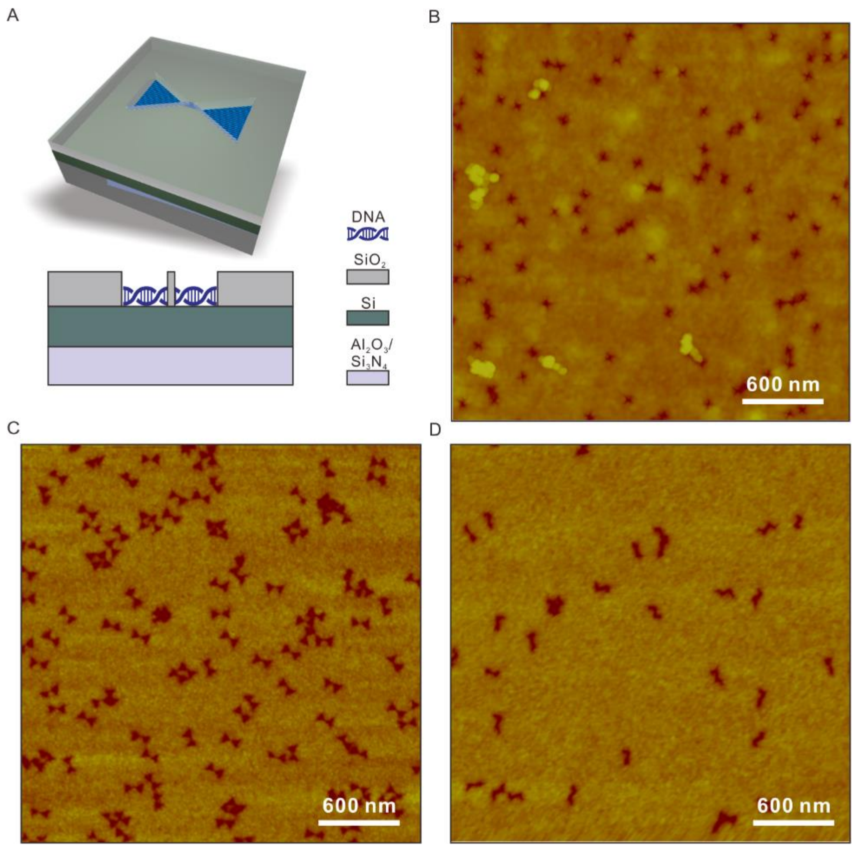

fig. S4. Fabrication of trenches/silhouettes with different DNA origami shapes. (**A**), Schematic view of the fabrication step with the BO-shape. (**B**-**D**), AFM micrographs of ST-, BO-, and CDL-silhouettes.

5) RIE etching of SiO_2 and Si

In order to deposit metal directly to the Si_3N_4/sapphire substrate, the Si layer between the SiO_2 mask and the substrate need to be etched through. RIE dry etching of Si was used for this purpose. As the selectivity of SiO_2 growth between Si surface and DNA origami is not perfect a thin layer of SiO_2 layer appears also on top of the DNA origami (this is also observed in ref. (*26*)). This thin oxide layer hinders the etching of Si underneath, and therefore a brief SiO_2 etching process is needed to reveal the Si film. The RIE parameters for SiO_2 etching are listed in table S3. To note, the etching time needs to be adjusted according to the SiO_2 thickness to maximize the preservation of DNA origami pattern.

table S3. Parameters for RIE SiO_2 etching.

CHF_3	25 sccm
Ar	25 sccm
Pressure	30 mTorr
RF power	100 W
Temperature	25 °C
Time	10-22 s

Dry etching of Si film is carried out immediately after the thin SiO_2 layer has been removed. The etching time was slightly longer than the time that is needed to remove the CVD grown Si thickness to ensure the exposure of substrate (etching parameters in table S4). At 30 °C, this dry etching process is isotropic, meaning that the etching is not only perpendicular to the surface, but also to the lateral direction. As an example, the undercut beneath the SiO_2 can be observed in fig. S5. This undercut is beneficial for lift-off process, but need to be taken into consideration when fabricate hollow structures.

table S4. Parameters for RIE Si etching.

O_2	8 sccm
SF_6	100 sccm
Pressure	90 mTorr
RF power	50 W
Temperature	30 °C
Time	35 s

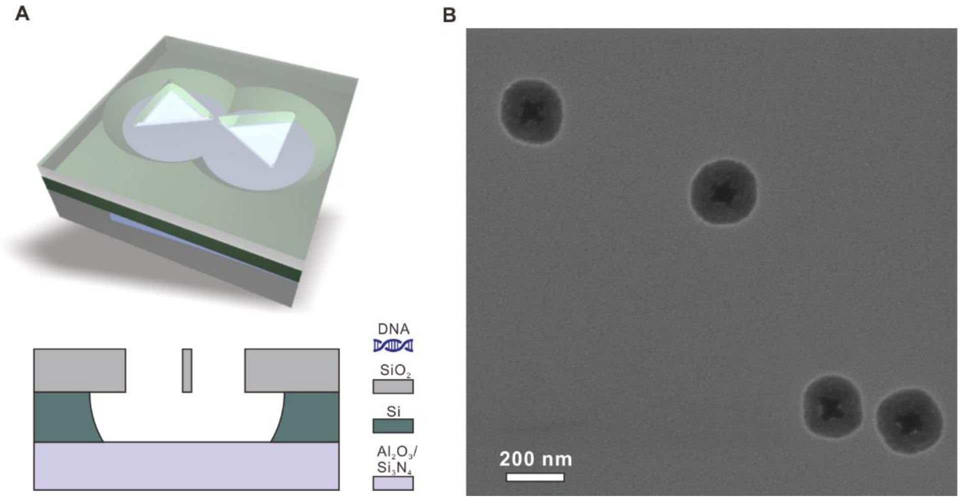

fig. S5. Isotropic RIE etching of silicon. (**A**)**,** Schematic view of the fabrication step with the BO-shape. (**B**)**,** SEM micrograph of a ST sample after the RIE etching of Si.

6) PVD (Physical vapor deposition) of gold

The metallic nanoparticles with the shape of DNA origami were formed by evaporating metal through the SiO_2 mask with origami-shaped openings (described above). In the utilized physical vapor deposition (PVD) process, i.e., evaporation, the sample and the target metal were placed in a ultra-high vacuum (UHV) chamber. The metal target in a carbon crucible was heated by an electron-beam gun while the evaporation rate was monitored by a quartz microbalance detector. An average rate of 0.05 nm/s and total thickness of 20 nm was used for gold deposition. Because the adhesion of gold to substrate was quite poor, sometimes a thin chromium layer of 2 nm thickness was evaporated before gold layer as an adhesive layer. The Au evaporation is carried out immediately after Cr deposition to prevent oxidation of Cr (Cr loses its function as an adhesive layer between the deposited gold and the substrate if it is oxidized). SEM images of typical samples after the metal PVD are shown in fig. S6.

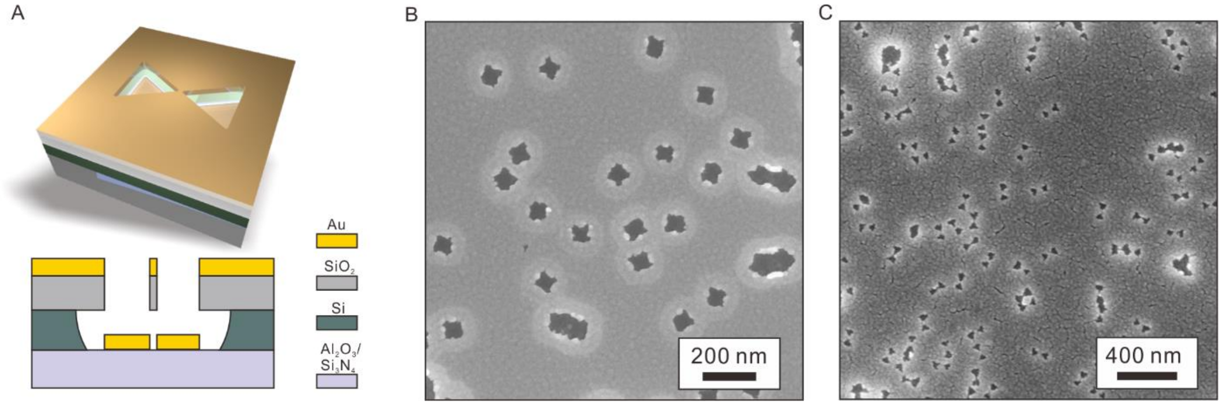

fig. S6. PVD of gold. (**A**) Schematic view of the fabrication step with the BO-shape**.** (**B**) Au on a mask with ST-openings. (**C**) Au on a mask with BO-openings (BO with gaps).

7) HF lift-off

To remove the SiO_2 layer (together with the metal film on top of it) and to reveal the formed metallic nanostructures at the sample substrate, various hydrofluoric-acid (HF) based etchants (38 % HF, 4 % HF, HF (38 %) : HCl (38 %) = 12:1) were tested. No significant difference was observed for the samples without the adhesive Cr layer. However, as HCl etches Cr, the mixture of HF and HCl cannot be used for the samples containing the Cr layer.

The sample was immersed in the HF-based etchant (in a plastic container) and gently stirred with plastic tweezers. Once the SiO_2 was etched and the metal layer was detached, the sample was rinsed with ddH_2O and isopropanol (IPA) and dried using a nitrogen flow. The lift-off is effortless for the samples having high particle density, since the etchant can attack the SiO_2 layer from each opening simultaneously. For the samples with fewer particles, longer etching time and ultra-sonication bath were needed to fully remove the SiO_2 layer. Because of the poor adhesion of gold, the pure gold particles hardly survived the ultra-sonication. Therefore Cr adhesive layer must be used for ultra-sonicated samples. After etching with HF, immersing the sample in IPA

leads to better ultra-sonication results, which is most probably because IPA can wet the small cavities under the gold film better. A typical sample after the HF lift-off is shown in fig. S7. The sample didn't have Cr layer, and consequently some of the particles have detached and left "empty" undercut circles.

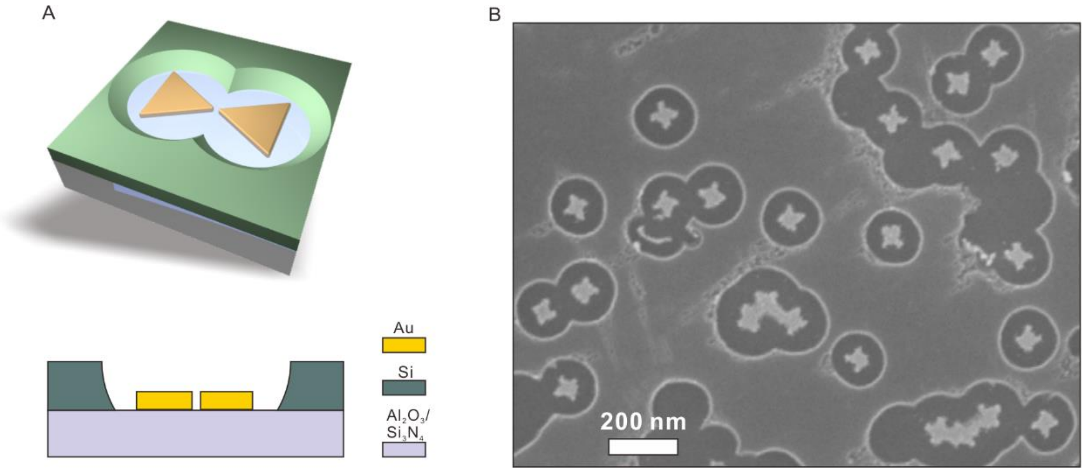

fig. S7. HF liftoff (removal of the SiO_2 mask). (**A**) Schematic view of the fabrication step with the BO-shape. (**B**) SEM micrograph of a ST sample after HF lift-off (the Si layer is not yet removed).

8) Post RIE cleaning of Si layer

The last step of DALI is to remove the spacing Si layer and leave only the metallic nanostructures on the substrate. A post RIE Si etching process can easily achieve the goal. The parameters of such Si etching were the same as in table S4, but the time can be slightly longer, e.g. 40 s, to ensure a proper removal.

9) Post fabrication analysis of heterogeneity of bowtie antennas

To estimate the overall homogeneity of the DALI-fabricated bowties, we determined the gap size, the thickness and the angle between the two triangles of numerous bowtie structures from a single fabrication patch.

The thickness profiles of the bowties were recorded by atomic force microscopy using a peak force tapping (tip radius = 2 nm) in air as shown in fig. S8. The mean roughness of the bowties' top surfaces was 1.9 ± 0.1 nm (Analyzed by Bruker AFM-analysis software). For the thickness analysis, we only included the data points within the triangles of the bowties. This was done by neglecting the edge points, which were more than the mean roughness from the average thickness of the analyzed bowtie. By this manner, we avoided the artifacts induced by a tip convolution, which extrudes and smooths the bowties edges. The average thickness for each bowtie was calculated and finally the mean of the average thicknesses of the bowties denoted as the mean thickness of the sample. The mean thickness of the analyzed fabrication patch was 21.6 ± 0.2 nm. The mean thickness and roughness indicate that the bowties are very similar in thickness and in a single bowtie, the local thickness differences are within 2 nm.

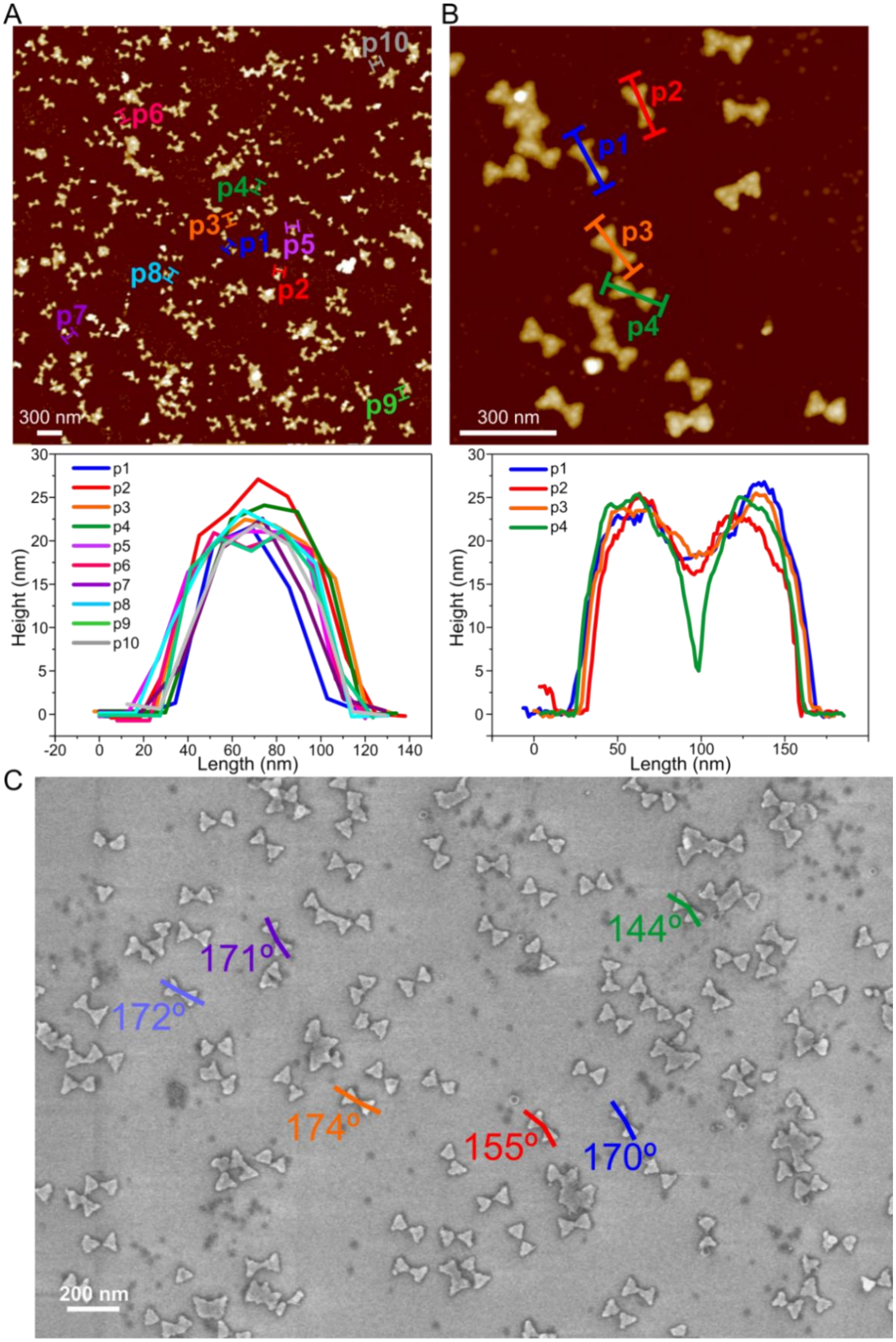

fig. S8. AFM images with the corresponding thickness profiles and a SEM image of Au bowtie antennas on a sapphire substrate. Examples of (A) widerange and (B) zoomed AFM images of bowties used for the thickness and roughness analysis. The corresponding thickness profiles are shown in the middle panel. Due to the tip convolution, the gap regions are not seen in all of the thickness profiles. (C) An example of a SEM image used in the gap and the bending angle calculations.

The angle and the gap size variations in the same fabrication patch were analyzed from figs. S8C and SA3 (lower) using ImageJ software. The statistical distribution of the bending angles had a peak value at 179° and distribution of gap sizes at 12 nm. Due to the nature of the variables, they both had an asymmetric distribution, which was fitted with an asymmetric Gaussian function, and the larger width (σ) was recorded as the standard deviation (SD). This yielded SD of 12º and 5 nm for the bending angle and the gap size, respectively.

note S2. Gap formation in a BO structure.

The key feature for a gap formation at the narrow bridge in a BO design is that the growth of SiO_2 is not purely anisotropic (perpendicular to the surface). During the growth process, the SiO_2 also grows on the walls of a freshly grown SiO_2 (see fig. S9). Due to the same reason the final metallic nanostructures have slightly smaller dimensions than the original DNA origami designs. The isotropic growth generates a thickness mismatch between the oxide layers on top of the narrow features and on top of the large open areas. Taking advantage of this fact, etching a certain thickness of SiO_2 layer away only exposes silicon layer beneath the larger areas but not beneath the small features. Therefore, a BO structure can contain a gap between the metallic triangles after Si etching and metal deposition (described in fig. S9).

Both the silicon oxide growth and the RIE etching of the oxide affect the gap formation. To ensure that a majority of the BOs have gaps, the oxide growth time has to be long enough to produce reasonable thickness differences in the oxide layer so that the following etching takes place through the triangle but not through the bridge. The gap size can also be roughly tuned by varying the etching time; however, it requires rather uniform oxide growth and RIE etching.

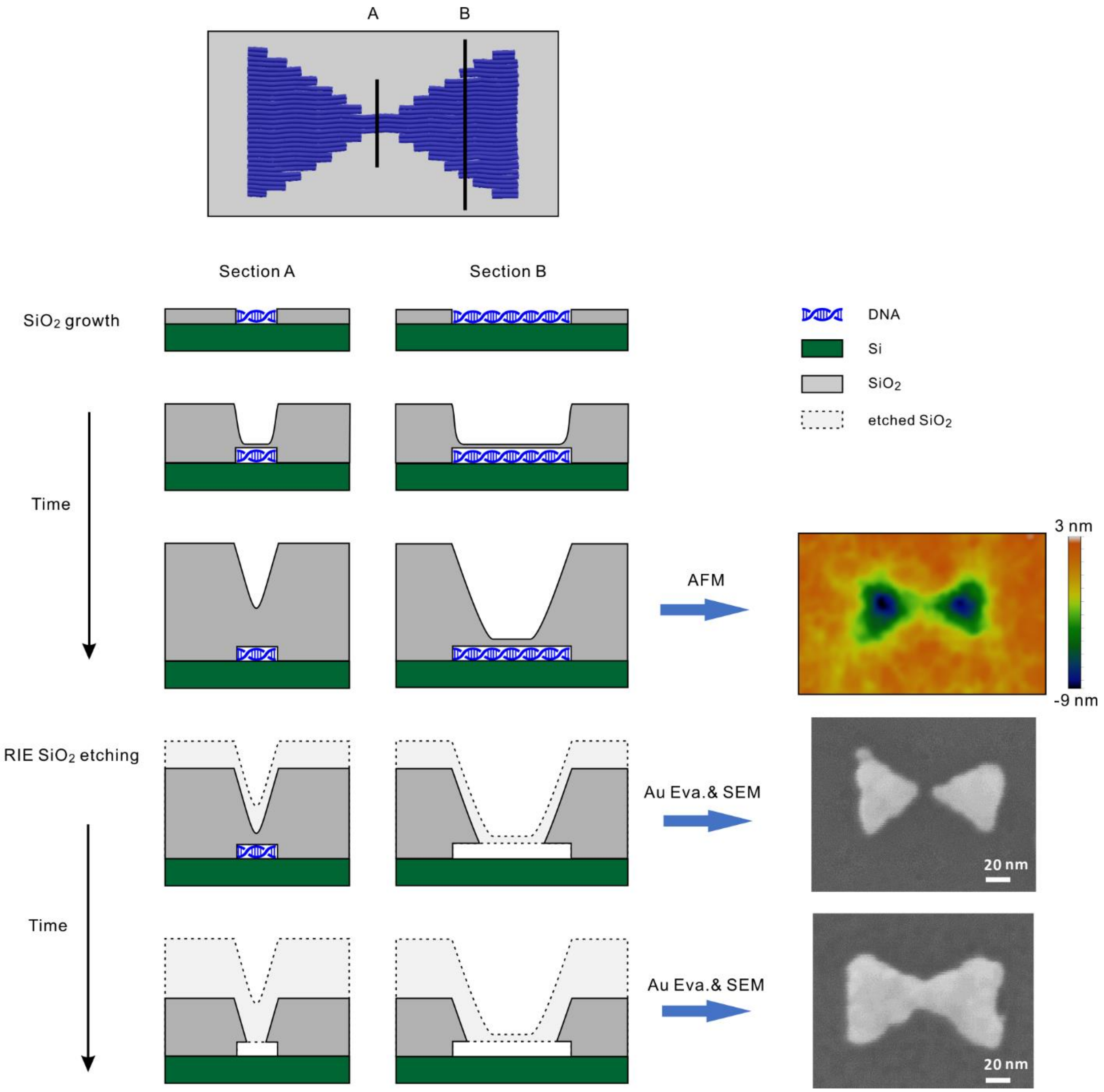

fig. S9. Schematic illustration of the oxide growth in the vicinity of the BO on a Si substrate. The thickness of the oxide above the DNA origami can be significantly higher at the Section A than at the Section B (same growth time). The AFM image on the right shows the thickness difference between the center of the triangles and the narrow bridge. After a short etching of the oxide only the triangles are exposed and therefore a metallic nanostructure with a gap is created. However, if the etching is carried out further, the whole origami area is exposed thus resulting in a full bowtie shape similar to the origami design.

note S3. Single-particle LSPR sample fabrication.

For the single particle LSPR characterization, the average distance between individual particles should be at least 5–10 µm to allow only one particle being encircled by an optical fiber spot. To satisfy this criterion, the samples with low particle density were prepared. The 20 nM DNA origami solution was diluted 2000 times using the deposition buffer to yield such particle separation. The final results may vary depending on the adsorption rate of origami onto the Si surface.

Besides low particle density, chessboard alignment grids were also fabricated on the sample to divide the surface into 200 µm × 200 µm areas so that the individual particles or patterns of a few particles could be easily identified and found. The grid pattern was created by UV-lithography using chromium mask on a glass slide after the SiO_2 layer was deposited (Step 4). Photoresist AZ4562 diluted with a thinner (1:3 w/w) was spun on the sample at 2000 rpm for 1 min. Then the sample was baked at 100 °C for 1 min. After exposure through the mask with the UV lamp for 30 sec, the sample was developed in water solution of AZ351B developer (1:4 w/w) for 2.5 min. Subsequently, the sample was etched by RIE using SiO_2 etching procedure (table S3) for 2 min, and finally washed in hot acetone to remove the AZ resist. The rest of the fabrication steps (Steps 5-8) were the same as for the samples with high particle density.

note S4. Single-particle linear polarization LSPR measurement.

In the single particle polarization measurements, we performed two calibration steps in order to determine the angle of polarization of the analyzer with respect to the orientation of the measured samples. The first task was to find suitable origami structures and analyze their orientation with respect to one of the grid axis. Then the polarization angle of the analyzer was aligned parallel to the same axis by using a polarizer.

To determine the angle of orientation of the metallized structures with respect to the marker grid and to the coordinates inside the grid opening, we used a dark field image of the area of interest combined with SEM images of the same area to identify potential candidates for the polarization measurements (fig. S10A). The dark field image was used to locate the interesting metallized origami structures, which were later imaged with SEM. Out of those structures the most promising ones were selected for the further study. For the LSPR measurements, a large-scale SEM image of the grid opening was taken, and by using that image and the SEM images of the targeted structures, it was possible to calculate the angles between one of the grid axis and the key axes of the structures. In the case of BOs, the key axes were along the gap and perpendicular to the gap. For the STs the key axes were along the arms.

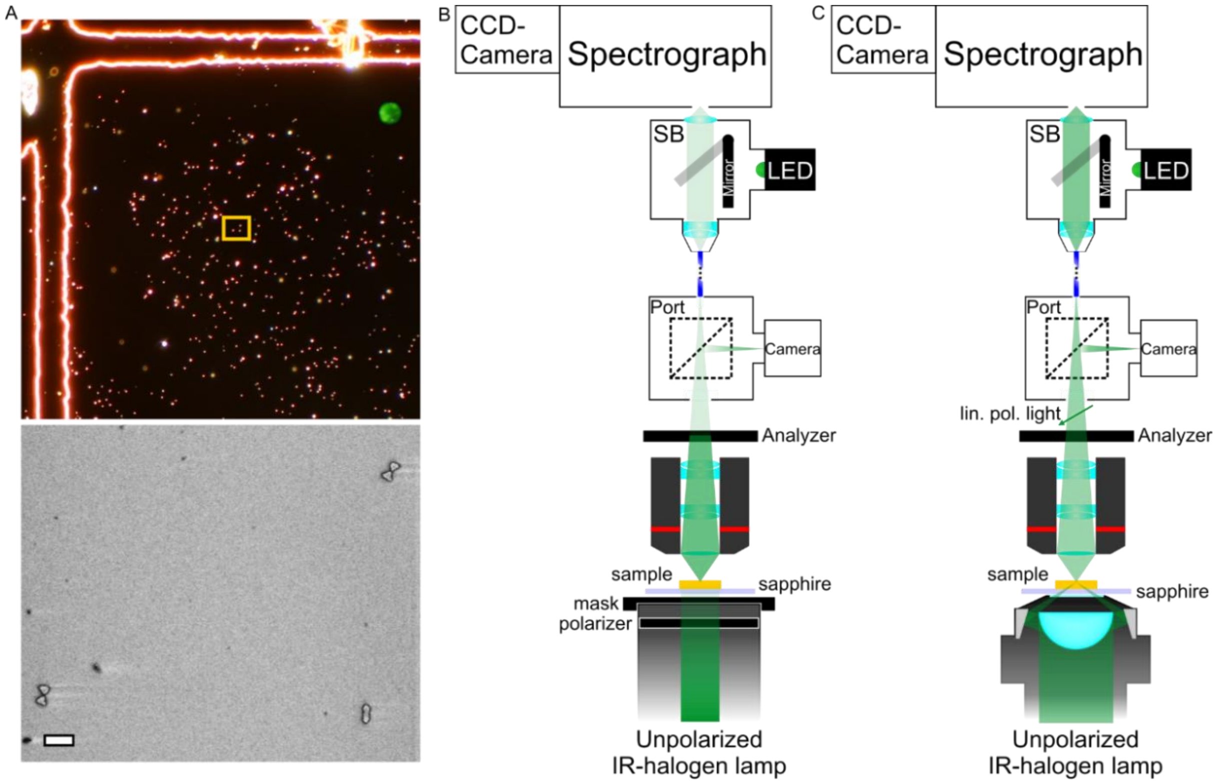

fig. S10. Schematics of the SPS setup. (**A**)**,** Typical dark field image of the metallized origami samples on a sapphire substrate. The green circle on the upper right corner is the image of the fiber spot. The SEM image below represents the area that is marked with a yellow rectangle in the dark field image. The scale bar in SEM image is 200 nm. (**B**), The schematic view of the SPS setup where the analyzer is aligned using the polarizer. (**C**), The schematic view of the SPS setup for measuring the polarization spectrum of the metallized origami sample.

The analyzer calibration was carried out in the following way. The used metallized origami sample with a marker grid (fig. S10B) was placed on a microscope slide with index-matched oil between the two. Then the slide with the sample was placed under the microscope and the sample and oil were left to settle for 0.5 h. A narrow, single slit mask was placed on top of the polarizer (fig. S10B) so that the slit was placed along the direction of polarization. Both the mask and the polarizer was then placed as close as possible to the microscope slide and turned in such a way that the slit opening was along the grid axis perpendicular to the axis that was used to calculate the angles of the targeted structures as described before. Then the analyzer was turned until a minimum signal was detected from the CCD-camera. The angle of polarization of the analyzer was then perpendicular to the polarizer *i.e*. along the desired grid axis. The polarizer and the mask were removed and a dark field condenser was switched to their place (fig. S10C). A drop of oil was placed onto the glass opening of the condenser and the condenser was pressed gently against the microscope slide until the vision came clear and bright in the oculars. The sample and the condenser were left to settle for 1 h, and a comparison picture was taken, since the sample could move during the settling.

After the oil had settled, the new sample orientation was confirmed by comparing images of the grid before and after the settling of the oil (note that any translational movement could be compensated by simply moving the sample to a new position and any rotational movement could be compensated by measuring LSPR spectra of the sample using adjusted angles of the analyzer). The analyzer was rotated to the angle that matched one of the key axes of the investigated structure. The fiber spot was then placed on the sample and the LSPR spectrum was recorded using the CCD-camera. The background was measured next to the particle on an empty spot. Then the analyzer was rotated 15° counterclockwise and the sample and the background measurements were carried out similarly as before. This was repeated until the angle was 180°, which was the last angle to be measured.

note S5. Additional single-particle linear polarization LSPR spectra.

This section contains additional polarization spectra of the metallized ST and BO samples. Polarization spectra of the two BO samples (fig. S11A and S11B) showed different kind of shift between the extreme peaks: the bowtie in fig. S11B has larger shift compared to the bowtie in fig. S11A. The cause for this is not exactly clear, but several factors influence the peak positions: the size of the gap, the angle between the triangles, the shape and the size of the triangles, sharpness of the triangles corners and uniformity of the thickness. When comparing the metallized BOs in main article Fig. 3 and the ones in fig. S11, it can be concluded that they have roughly the same size and have roughly similar roundness in the corners, but the angle and the gap between the triangles is varying. The peak in the perpendicular direction is roughly constant in all of the samples, but the peak associated to the gap mode is red shifting from 704 nm (Fig. 3A) to 814 nm (fig. S11B). It has been reported in literature (*39*), that, up to a certain small angle, higher the angle shift from the case where the triangles are facing perfectly each other more redshifted the LSPR peak is. On the other hand, if triangles are close to each other, then the LSPR peak will blueshift when the gap between triangles is increased (*40*). In our case, even though the angle shift from 155° (Fig. 3A) to 173° (fig. S11B) would suggest blueshift, the redshift is most probably due to smaller gap in fig. S11B when compared to fig. S11A and Fig. 3A.

For the ST samples, it was discovered that some of the STs had the extremes of the LSPR peak along the arms (fig. S11D and S11E) and other between the arms (fig. S11C and S11F). The exact reason for this is unknown, but there are a few possible factors that may affect. It is possible that the STs actually have disc-like qualities due to their roundedness. This would cause the peak extremes to be located along the elliptical axes of the rounded, disk-like cross rather than along the arms. The ST-shapes in figs. S11D and S11E have well defined arms but the length of the arms is different compared to the ST in fig. S11F that has rounded corners. Other possible explanation is that the STs are not exactly flat but rather have bumps and grains, which might have considerable influence on the optical properties of the sample. The peak extremes in

figs. S11C-F are 682–741 nm, 646–677 nm, 655–690 nm and 647–682 nm in the corresponding order.

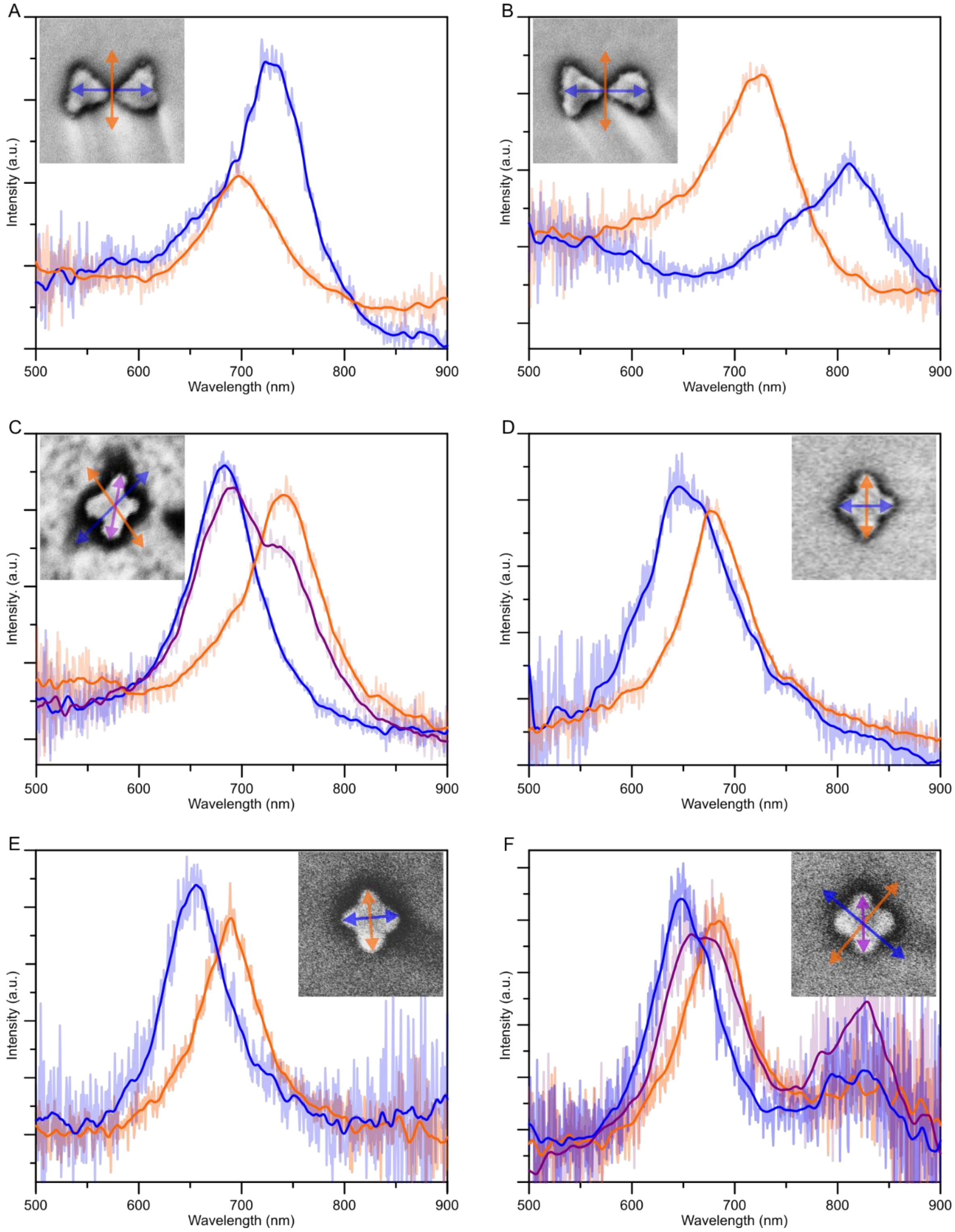

fig. S11. Single-structure spectra of different metallized origami shapes. (**A**),(**B**) The spectra from two different BOs (corresponding BOs shown in the insets, size 200 nm x 200 nm). (**C**)-(**F**) The spectra

of four different STs (corresponding STs shown in the insets, size 200 nm x 200 nm). The color of the arrow (polarization direction) in the insets corresponds the color of the measured spectrum.

note S6. UV-Vis measurement of CDL samples.

Besides the CD spectra, UV-Vis spectra were also obtained for the CDL samples (fig. S12). The S configuration sample and the random orientation sample showed similar absorbance spectra which proved the difference of CD spectra was not caused by the different coverage of particles on the substrates or other non-chiral properties.

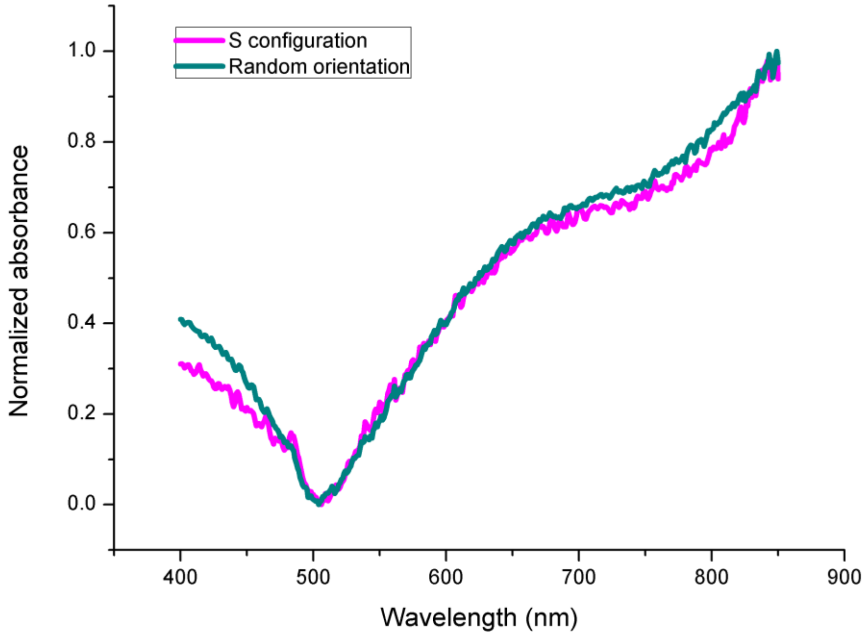

fig. S12. Normalized UV-Vis spectra of CDL samples with S-configuration and random orientation.

note S7. Numerical simulations.

The optical properties of the fabricated structures were simulated by the finite element method (FEM). All numerical calculations were carried out with the RF module of the Comsol Multiphysics 5.2 modeling software. The absorption, scattering and extinction cross sections were calculated for single gold particles placed on a substrate with a refractive index of n_b=1.76, corresponding to the sapphire substrates used in the experiments. The wavelength dependent complex refractive index for evaporated Au from Olmon et al. was used for the particles (*41*). The medium refractive index above the particles was set to n_a=1. A normally incident linearly polarized plane electromagnetic wave, with a 500–900 nm wavelength was used for the BO and

ST particles. For the calculations of circular dichroism (CD) of the chiral particles (the S- and Z-type) normally incident circularly polarized light with wavelengths from 500–1100 nm were used. The CD spectra were calculated as the difference between extinction cross sections of clockwise and anti-clockwise polarized light or CD = $\sigma_{eACW} - \sigma_{eCW}$.

Figure S13 depicts the simulation geometry for a CDL-particle (S-orientation) with the perfectly matched layers surrounding the physical domains and a clockwise polarized incident light. The electric field vectors (red) and wavevectors (black) are shown for the incident light with a wavelength of 640 nm. The maximum and minimum mesh element sizes were set to 50 nm and 1 nm respectively.

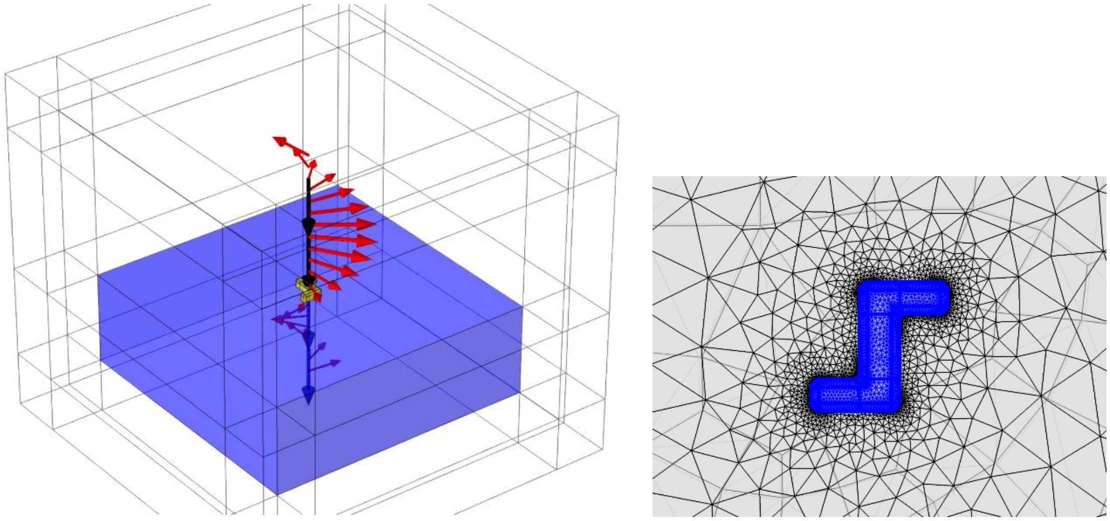

fig. S13. Simulation geometry for a CDL particle (S-shaped orientation) with a clockwise polarized incident light and used mesh. Perfectly matched layers surround the physical domains. The electric field vectors (red) and wavevectors (black) are shown for the incident light with a wavelength of 640 nm. The right picture shows the used mesh in detail.

Geometry of the simulated particles

The BO is composed of two equilateral triangular prisms with rounded corners and rounded upper edges. After filleting the corners and edges with a radius of 5 nm the thickness of the rounded corner triangle base was h = 52 nm and the tip to tip side length was l = 57.1 nm as shown in fig. S14A-C. The gap size between the triangles was 6 nm. The thickness of the bowtie structure is 20 nm. The simulated LSPR peaks can be seen in the article Fig. 3 and in the fig. S15 (Black).

The ST particle is composed of two rounded corners and overlapping boxes with slightly different lengths. The arm lengths and rounding of the corners were chosen by fitting the measured and calculated extinction spectra. The length of the long axis of the particle was 71.5

nm and 64.2 nm for the short axis. The width of the arms was 25 nm and the thickness of the particles was 20 nm. The outer and top edges were rounded to a radius of 8 nm and the edges in the inner corners were rounded to a radius of 5 nm. The lower edges in contact with the substrate were not rounded. The geometry of the plus shaped particle is depicted in fig. S14D-F.

The geometry for two different CDLs is shown in the fig. S14G-I and fig. S14J-L**.** The symmetrical S-shaped CDL (G-I) was composed of three rounded-edge boxes. The long axis was formed by a box with the length of 80 nm and the width of 25 nm. The short axes were formed by equal-sized boxes of a length of 55 nm and a width of 20 nm. The thickness of the particles was 20 nm and the corners and edges on the top of the particle were rounded to a radius of 5 nm.

The asymmetric CDL depicted in fig. S14J-L was composed of three different sizes of overlapping boxes. The long axis of the particle was composed of a box 80 nm long, 25 nm wide. The longer arm of the particle (depicted in the upper part of the fig. S14K) was formed by a box with the length of 50 nm and the width of 20 nm. The shorter arm was composed by a box with the length of 40 nm and the width of 20 nm. The thickness of the particle was 20 nm. The corners and edges were rounded to a radius of 8 nm (outer edges/corners) and the two inner corners were rounded to a radius of 5 nm.

Simulating the plasmonic properties of the particles with varying geometries

To reveal the effect of the observed fabrication heterogeneity (See Note S1/9), we simulated several structures, which were deformed by the amount of the observed standard deviation (SD). To account for the varying bending angle, we simulated a BO geometry with one of the triangles tilted in-plane by SD (12°) while not changing the gap apexes. This bending yielded no noticeable effects on the LSPR modes or the field enhancement, as can be seen in fig. S15 below. The gap size and the thickness were also separately increased corresponding to the variations in the fabrication process. The gap size was increased by the amount of relative SD (gap size increased from 6.0 nm to 8.3 nm (38% change)) and the thickness was increased by the mean roughness (2 nm, i.e. thickness was increased from 20 nm to 22 nm). The increased thickness and gap size induced 8.5 and 12.5 nm blue shifts in the LSPR gap mode, and decreased the maximum field enhancements by 12% and 30%, respectively (See fig. S15). The wavelength of the perpendicular LSPR mode was only affected by the increased thickness, with a 6.5 nm blue shift, but all the variations yielded a small decrease in the field enhancement of this mode. All the changes in the plasmonic properties due to the variations in the fabrication process were considered minor.

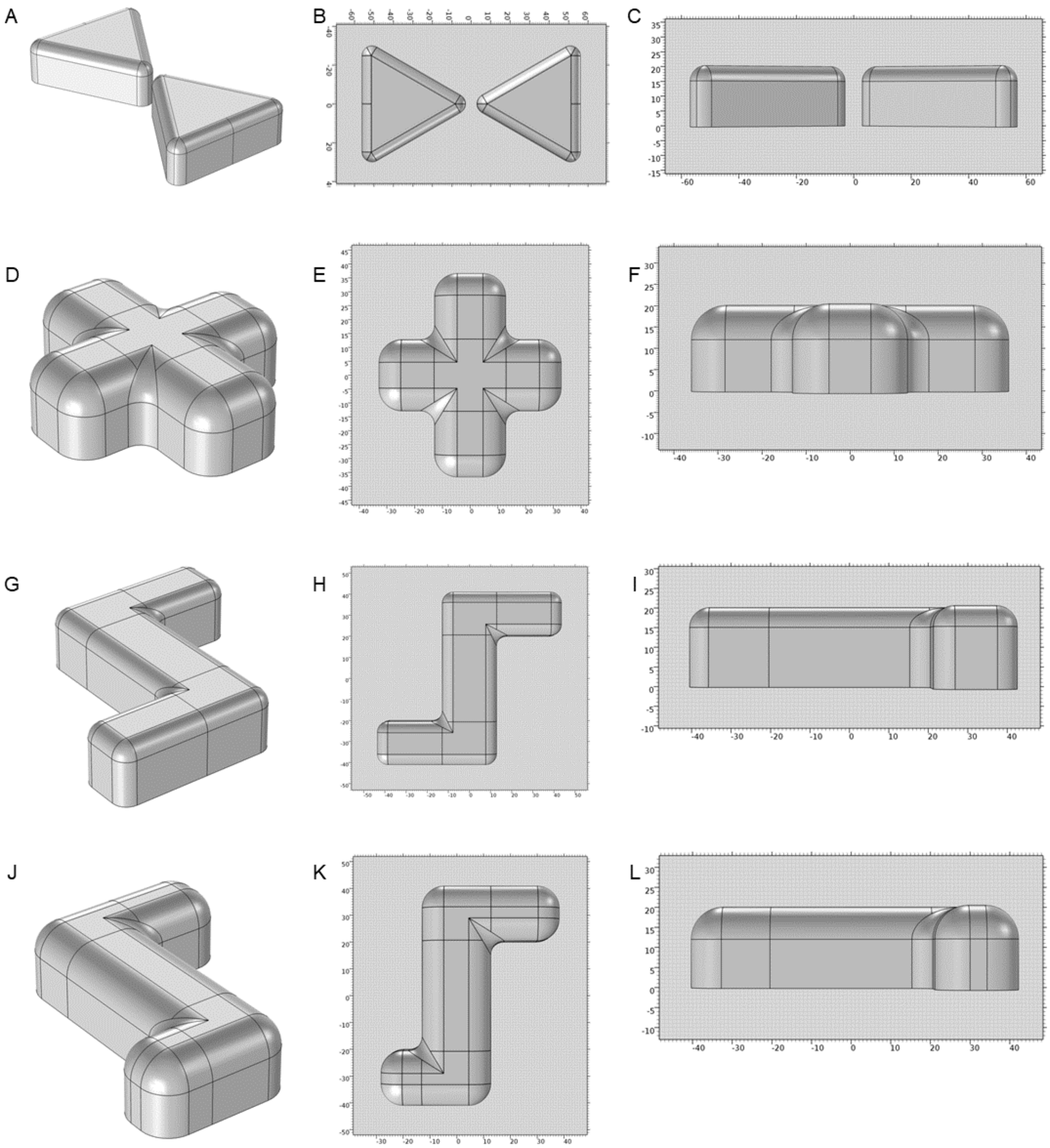

fig. S14. Geometries of the different types of particles for the Comsol simulations. (**A**)-(**C**) BO structure. (**D**)-(**F**) ST structure. (**G**)-(**I**) symmetric S-shaped CDL. (**J**)-(**L**) asymmetric S-shaped CDL.

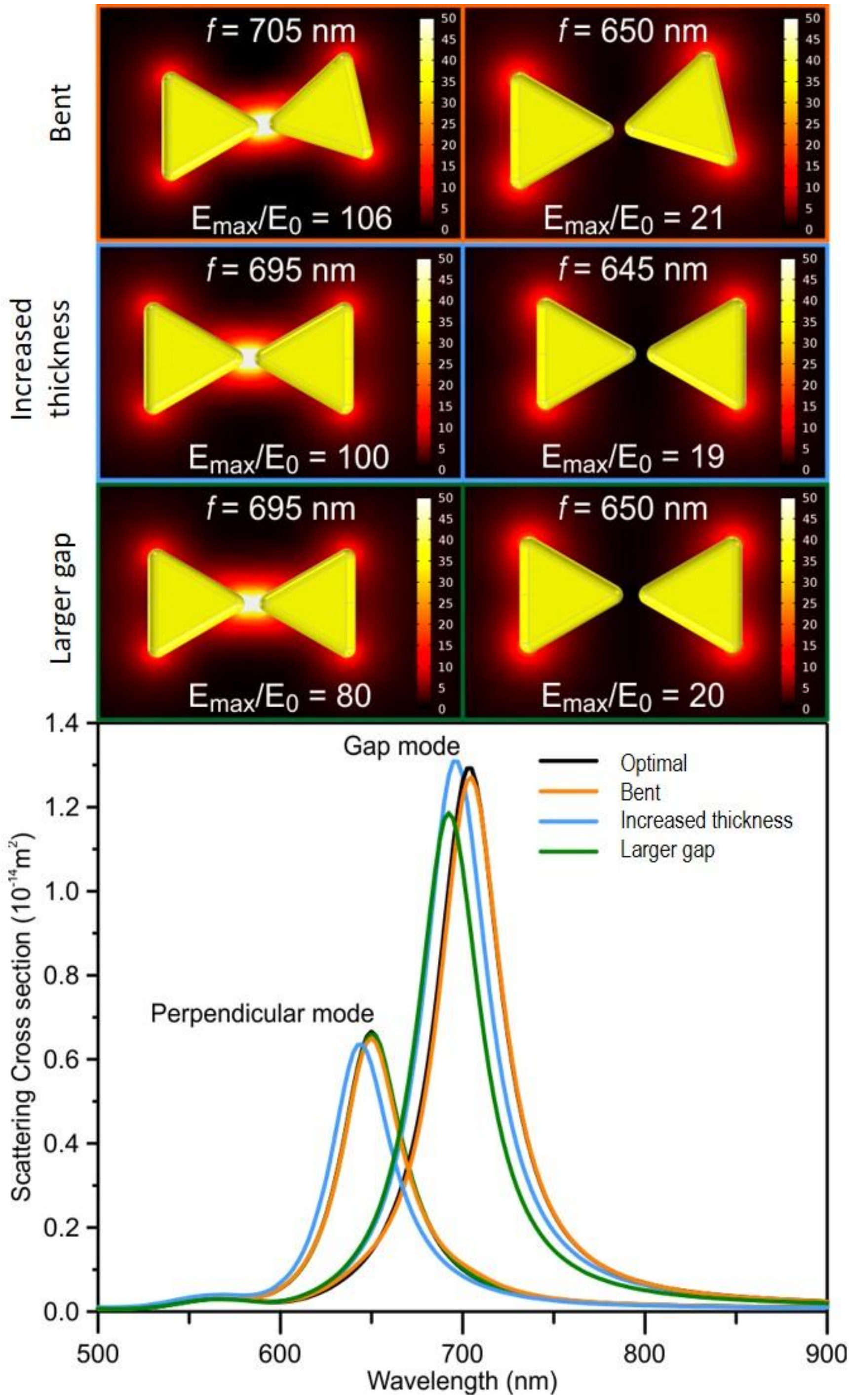

fig. S15. Simulated LSPR spectra and field enhancements (*E*/*E*$_0$ at resonance frequency) for the optimal bowtie structure and for the structures with geometries altered by the amount of the observed SDs. Bottom graphs: The black curves correspond to the bowtie in figs. S14A-C and article Fig. 3 (both the perpendicular and the gap mode). The orange curves represent a bowtie structure bent by 12° from the symmetric 180°, while the blue curves indicate a thicker bowtie structure (20 nm increased to 22 nm). The green curves correspond to a bowtie structure with a larger gap size (from 6.0 nm to 8.3 nm). The perpendicular mode is affected only by the change in the thickness, while the gap mode is affected by the changes in the gap size and the thickness. Top contour plots: Simulated field enhancements, E/E_0, for all the deformed shapes and both the LSPR modes. E_0 is the incoming field.

Appendix

Design and sequences of BO

caDNAno design of BO

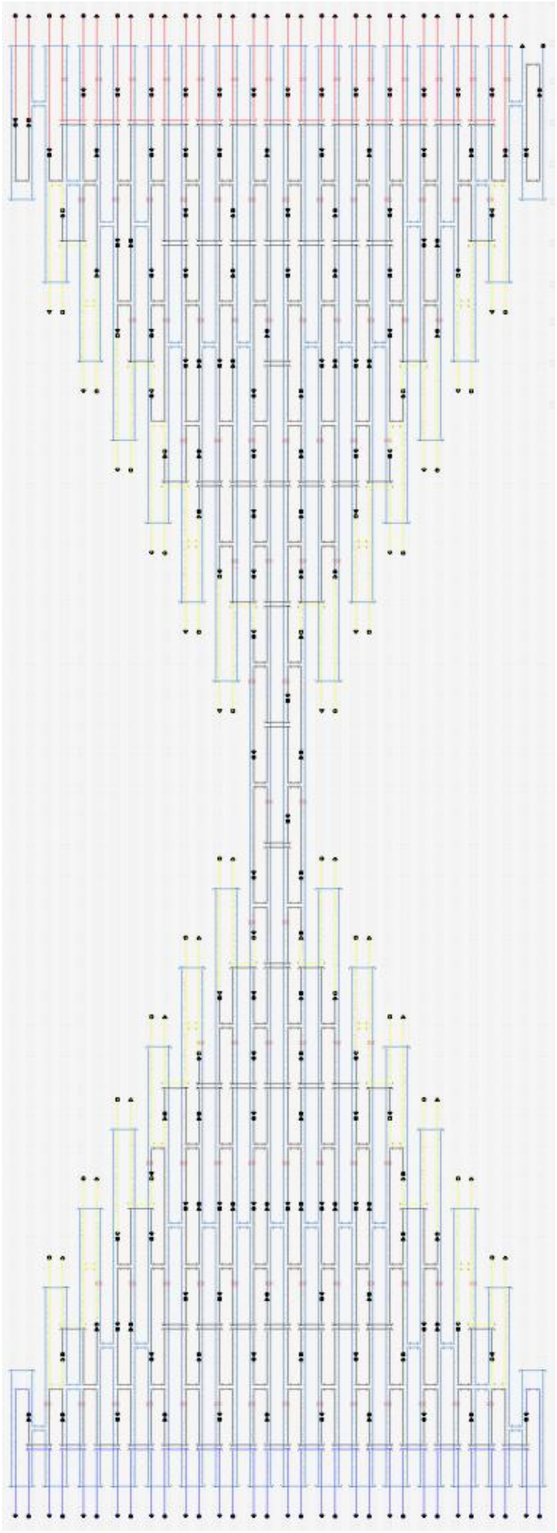

Staple strand sequences (5' → 3') for BO (in total 205 staples)

Core staples (black, 118 staples):

CGGAAATTATCGCGCAGAGGCGAATTTAGGAGCACTAACATTTGAGGG
GCATTTTATGCGATTTATACCACATTCCTATC
CCTTTTTAGCTTAGATTAAGACGCAAACAGTACATAAATCCAAGAAAA
GGGCCTCCCGGAAACCAGGCAAAAGGTCACG
CCTGTATATCCAGAAGCCAACAGAGAGGGTA
CAACAGTTATGATGAAACAAACATAATATATGTGAGTGATGAAAACA
CCGGAAGCGACTTCAAATATCGCAGCGGATTGCATCAAAATCAGGT
AATCAAGTGAACCTACCATATCAAGGAACAAAGAAACCACTAATTCGC
CCAGCAAAACCTTTTACATCGGGAAGTATTAGACTTTACATTGACCGT
AAGTTTAGAACCCTTGTGAGGCGGTCTCGGC
GTCTGGCCAAAAACAGGAAGATTGTAATCGTA
TTATTACGCAGTATGTTTCAACCGATTGAGGG
TCGATAGCAACAGAAATAAAGAAATTTAAAAGTTTGAGTATCATCAAC
CTCAGAGCGCCACCACCGGAACCGAGCGACAG
AGCGTCAGATCACCGGAACCAGACGCCACCAGAACCACCAAATCCTC
TGAGGCTAAAACGAAAACCTGCTCCATCCCCC
GCATCGTAGGCACCGCTTCTGGTGTTCGCTATTACGCCAGAAACGACG
AAAGGAACTAATGCAGCCAGAGGGGGTGAGTA
AAAGGTGAAGACAAAAGGGCGACATAGCAAACGTAGAAATAATAACG
GAATTAACAAAAATGAAAATAGCATAAACAG
ATTAAGAGCTTTTGCGGGATAAGTTTTAACGGGGTCAGTGCTATTAT
CCTCAATAAATCAAAAAAGATTAAGAGATTTG
TCCGGTATAATCAATAATCGGCTGCGAGCATGTAGAAACCTGTTTATC
GGCTCATTTTCAACTTTAATCATTTACCCAAATCAACGTTTCATCAA
GTCCACTATGGACTCCAACGTCAAAGGGCGAA
TAGCGATAACCTCCGGCTTAGGTAACGCGAGAAAACTTTAAGGCGT
AAAATACCACAATATTTTTGAATGTTGGCAGATTCACCAGACGCTCAT
TCAGCGGACCACAGACAGCTGTAATTTTCCC
AACACTGAGCCCAATAGGAACCCATGAAAATCTCCACCCT
TATATTCGTAGTTGCGCCGACAATGCTTTCGAGGTGAATTCCAAAA
AATTCCCGGATATTCATGTGAATTACCAAATT
CCGGAATCCAATCGCAAGACAAAGTGGGTTATATAACTATTTTCCCTT
CAGACGATTGGCCTTGATACAGGAGTGTACT
TTCTTTTGCTGCATTAATGAATCCACATTAATTGCGTTCCTTGCTG
AACAGCTCGTTAGAATGTTTTTATAAGTCGG
GATACCGAGTCGCTGAGGCTTGCAATCGGAACGAGGGTAGTTAAACGG
CATTTGGGATAGAAAATTCATATGGCAACAT
TGTAATTTCAGTATAAAGCCAACGCGACCGTG
GACAACTAATATACAGTAACAGTATCACCAGTAGCACCATTACCAT
ATGTGTAGGTAAAGATTTTTGAGAGATCTAC

TGGCACAGGAACGAACCACCAGCCACGCTGAGAGCCAGCGGCAAAT
AAGACAGCGGGAGTTAAAGGCCGGCTGAGACTCCTCAAGAGAAGCTT
GGTTTAATATACCAGTCAGGACGTGGTAGAAAGATTCATCGGAATTAC
TCACCGTATGCTCAGTACCAGGCGATGAAAGT
AGGGAATTAATTAATATGTAAATGCTACTCC
ACCGAGCATAAAGCTAAATCGGTGTTTAGACTGGATAGCGCAAAAGA
TAGCAAGGGGTTTAACGTCAGATGCGTATTAAATCCTTTGCCGTCGGA
AAACCAAAGTAAGAGCAACACTAGAACAACATTATTACATGGGAAG
GTAGTGAAATTGTTATCCGCTCACAATGAGTG
GTAGCTATTCAAAAGGGTGAGAAATTTTTAGA
ACTATCGGGCGCTCACTGCCCGCTTTCCATCAGTGAGGCCCTTGCCT
GCGTTTTCTGGATTATACTTCTGAATCATCATATTCCTGAATTTTTA
ACCAATAGAATTGTAAACGTTAATGTCTGGAGCAAACAAGCGGAGAGG
GGTAATCGTCACCCTCAAGGCACCAACCAGGT
TTCCGAAAGCGAAAATCCTGTTTGACGTGCTTTCCTGATTGCCCTTC
TTGGTGTCATTTGAGGATTTAGAGAAACAATAACGGATACTTGAGC
CTCAGGAAGATCGCACGTGCTGCAAGGCGATT
GGATTAGCGGGGTTTCTCAGGAGGTTTAGTA
CTTTACCCTAAACAGTTCAGAAAATCGTCATAAATATTCGACGATAA
TTCAATTAAATCGTCGCTAGGTAAATATTGA
ATAAGGGACATAGGCTGGCTGACCAACAAAGCTGCTCATTCTTGAGAT
CAGCGATTCATGAGGAAGTTTCCACAACGGCTACAGAGGATAACCGA
GCGTCTTTCCAGACAGAACGCGCCCCTTAAATCAAGATTGCGGGAGG
TTTTCATCGTAGGAATCCCAATAGCAAGCAAA
GCCAGCTTACATTATCATTTTGCAATTATTTGCACGTAAAGCACCG
TCTGCCAGACTAATAGATTAGAGAATACCAAGTTACAAAATTCATT
CAAAATTACAATATCTGGTCAGTTAGCAAATGAAAAATCTCGCCATTA
TCAAAAACAGAAGGAGCGGAATTATAATGGAAGGGTTATTGCCTTT
TTATTAGCGTTTGCCAGCATTGACAGGAGGT
GAAACCTGTCGTGCCACACCAGTGAGACGGGC
AACAGTGCTACATGGCTTTTGATGATATTCAC
AGAATCCTATAACCTTGCTTCTGTACCTGAGCAAAAGAAGGAAAGGAA
TGAGAAGTCAGAGCGGGAGCTAAATTGACGAGCACGTATATAACCACC
GAGTAATGGAACGAGGCGCAGACGTGTCGAAATCCGCGGAGGCAAA
AGCGCATTTTTAGTATCATATGCGAATAAACA
AGAATACACGTAATGCCACTACGAGCAGCGA
CTGAACAATAAGCCCAATAATAAGAGCAAAAAAAGCCTGAGACGGGA
AGCCTTTTTGCTCTCAACATGTTTAAACTCCAACAGGTCGAACCAGA
GGAGCCTTAATAATTTTTTCACGTGTGAGAATAGAAAGGCAACAGTT
AGTTTTGATACATAACGCCAAAAAGTTGAGATTTAGGATAAGAACT
GAGTAGAACATTGCAACAGGAAAATCACACGACCAGTAAAGAATACG
AAACAAATACCAGAGCCGCCGCCATCTTTTCATAATCAAAACTGTAGC
GTAAAATACTAAAACACTCATCTTCATCGCCTGATAAATTGGTCAATC

CCGCCAAAAAAAAGGCTTCTTAAACAGGATTA

TAAATAAGTTATACAAATTCTTACAGGCAGAGGCATAAAAACAGGGA

ATTAAATGTGAGCGAGTAACAACCCCGAACGT

TGATAAATTTCAAATATATTTTAGAGAGACTA

TTAAGGATGCAAATCATAATTACTAGGAAAC

AAACCGTCAAGGGAAGAAAGTTTGGAACAAGA

AAAGCGTATAAAAGGGACATTCTGCAATATTACCGCCAGCGAACTCAA

ACCAGGCGACCGAACTGACCAACCAACGGAGATTTGTATTGACCCC

GAATACCCCTATCTTACCGAAGCCCCACAAG

TTTTCGAAGCCCGAAATTCCCAATTCTGCGAATCCATATA

AACAATAGACAATAAACAACATGTAGTAATAAGAGAATATCGCCAACA

ATTTGCCAGTTACAAAAGCCTTTACAGAGAGA

ACGCCAGGGTTTTCCCATGGTCATAGCTGTTT

ATTAATGCAGAATCGATGAACGGTATAAGCAAATATTTAGAACGCCA

AAACATTAAGGCAAAGAATTAGCAAGGTGGCA

GGAAATACATTAGTAATAACATCAACCGAGTAAAAGAGTCCAGAATCC

CATAGGTTACTTAGCCCTTGACAAGAAATCAA

GGGCGCGAGCTGAAAAAATTAAGCAATAAAG

ATAACATTTTCGAGCCTCAGCTAATGGCCTA

TATAATCCTGATTGTTATCGGCATTTTCGGT

ACGTTGTACTGGCGAAAGGGGGATTCCAGCCAGCTTTCCACCGTGCA

AGGTCATTGCCTGAGAATTTTGTTAAAATTC

GAGGCATAATAGCGAGAGGCTTTTGTCCAATACTGCGGAACGAGAATG

ACACCCGCGCGGTCACGCTGCGCGATAGGGTT

CGTCACCGTCGCCTGATTGCTTTGCCGTCAATAGATAATAAGATGGGC

TTTGTTAAATCAGCTCTTATCAGATGATGGC

GACGACGACAGTAAGTATTAACAGGTTATCTAAAATATCTTATTCAT

AAAAATCTCGAGTAGTAAATTGGGCAGTGAATAAGGCTTGTGTACAG

TTGAGGAACCGCCTGCAACAGTGCAGAAGATAAAACAGAGCTGACCTG

TTCTCCGTGGGAACAAACGGCGGAAACAATTC

TAATACTTATATTTTAAATGCAATGCCTAATAGTAAAATTGTACCAA

AATGAAATAGCAATAGAAAAGAACTGGCATGA

AAACGCAAATACATACATAAAGGTGGTTTACCAGCGCCAAATTATCAC

TATTAATTTGCGTAGATTTTCACCGGAAACGTCACCAATGAAACCA

CACAACATGCTCGAATTCGTAATCAGTCACG

Sidestrands (left) with poly-T overhangs (blue, 17 staples):

TTTTTTTTGAAACAAAGTATTTGAAAGAGGTTTTTTTT

TTTTTTTTATCGCCCACGCCTTTGAGGACTTTTTTTTT

TTTTTTTTAAAGACTTTTTATACCAAGCGCTTTTTTTT

TTTTTTTTATTTTTGCGGATGGCTTAGAGCTTAATAATGCTGT

TTTTTTTTACGAACTAACGTCATAACCCTCTTTTTTTT

TTTTTTTTGAATTGCGAATTAATTGTATCGTTTTTTTT

TTTTTTTTAAACACCAGAAACGTTAATAAATTTTTTTT
TTTTTTTTGTTTATCAGCTTGACAACAACCTTTTTTTT
TTTTTTTTAGCTTCAAAGCAGGATTAGAGATTTTTTTT
TTTTTTTTGTCAGAAGCAAGTTTTAATTCGTTTTTTTT
TTTTTTTTAAACAACTTTAACAACTAAAGTTTTTTTT
TTTTTTTTGTACCTTTAATGATAAGAGGTCTTTTTTTT
TTTTTTTTGTTTACCAGACATTGAATCCCCTTTTTTTT
TTTTTTTTTTAGTAAATGATGGGATTTTGCTTTTTTTT
TTTTTTTTCTCAAATGCTTTGACTATTATATTTTTTTT
TTTTTTTTACAGATGAACGGCCCTGACGAGTTTTTTTT
TCATAGTTTCTAAAGTTTTGTCGTCTTTCCAGACGTTTTTTTT

Sidestrands (right) with poly-T overhangs (red, 30 staples):

TTTTTTTTGCTAGGGCGCTGG
TTTTTTTTATAATATCCCATC
TATTTACAGCTATTAGTCTTTAATGCGCTTTTTTTT
CAAGTGTACGCGCTTAATGCGCCGCTACTTTTTTTT
TCAGAAGTACCGCACTCATCGTTTTTTTT
TAGGTCTGTTAATTTCATCTTCTGACCTTTTTTTT
TTTTTTTTAGGGCGCGTACTA
TTTTTTTTCTCAAATATCAAA
TTTTTTTTTTTATCAAAATCA
TTTTTTTTGAACTGATAGCCC
CCCTCAATATTACATTTAACAATTTCATTTTTTTT
TTTTTTTTAACGGGTATTAAACCATATAGAAGGCTTA
TTTAACAAAAAGTACCGACAAAAGGTAATTTTTTTT
CGGTACGCTGTCCATCACGCAAATTAACTTTTTTTT
AGACGACGATAAGTCCTGAACAAGAAAATTTTTTTT
TTTAATGGTGAGAAGAGTCAATAGTGAATTTTTTTT
TAAAACATAAAGCATCACCTTGCTGAACTTTTTTTT
TGAAATACCTCAACAGTAGGGCTTAATTTTTTTTTT
TTTTTTTTAGTAATTCTGTCC
TTTTTTTTTTTTAGACAGGAA
GAGTGTTGTTCCAGCGAAAGGAGCGGGCTTTTTTTT
CTTCTTTGCTACATTTTGACGCTCAATCTTTTTTTT
TTTTTTTTAAATTTAATGGTT
TTTTTTTTGTCTGAAATGGAT
TTTTTTTTGAGAATCGCCATA
TTTTTTTTTTGAATTACCTTT
TTTTTTTTCGTTGTAGCAATA
CTAATTTATCTTTCCTTATCATTCCAAGTTTTTTTT
TGGTTGCTCAGGAGGCCGATTAAAGGGATTTTTTTT
TTTTTTTTAGAACAAGCAAGCCGTTTTTA

Sidestrands (tilted edges) with poly-T overhangs (yellow, 40 staples):

TTTTTTTTGCGCAACTGTTGGGAAGGGCGATCGGTGC

TTTTTTTTAGAGCCACCACCCTCACAGAACCGCCACCCTCTTTTTTTT

AATTGAGTAGTCAGAGGGTAATTGAGCGCTAATATTTTTTTTT

TTTTTTTTGTTAATGCCCCCTGCCTATTTCGGAACCCTTGAGT

TTTTTTTTCCTGAATCTTACCAACCCAGCTACAATTTTATTTTTTTTT

TTTTTTTTAAGGATAAAAAGGCCGGAGACAGTCAGCTGATAA

CCATATTATTTATCCCAATCCAAATAAGATTTTTTTT

TTTTTTTTCCTTATAAATCAAAAGAATAGCCCGAGATGGTGG

TTTTTTTTTCAGAACCGCCACCCTCAGAGCCACCACC

AAACTAGCATGTCAATCATATGTACCCCGTTTTTTTT

TTTTTTTTAGTACAAACTACAACGCCTGTAGCATTTGTACCGT

TAATCAGTCCTCCCTCAGAGCCGCCACCCTTTTTTTT

ATTAAAGGTTCCAGTAAGCGTCACCGTATAAACATTTTTTTT

GCCAGTGCGATCCCCGGGTACCGAACGAGCCGGAATTTTTTTT

ACCGCCTGTGGTTTGCCCCAGCAGTCGGCAAAATCTTTTTTTT

TTTTTTTTGGTTTGCGTATTGGGCGCCAGGGTGGTTT

TTTTTTTTATATTCAACCGTTCTAAATCACCATCAATATGTTTTTTTT

ATAAAAGAAACGCAAAGACACCACGGAATTTTTTTT

TTTTGAAGTCTAAGAACGCGAGGCGTTTTAGCGAATTTTTTTT

TTTTTTTTAGCAAGCGGTCCACGCGCCCTGAGAGAGTTGCTTTTTTTT

TTTTTTTTCCTCCCGACTTAGTTGCTATTTTGCACGCTAACGA

ACCCTCATTTGCGGGAGAAGCCTTTATTTCAACGCTTTTTTTT

AGCTAACTGGCCAACGCGCGGGGAGAGGCTTTTTTTT

TTTTTTTTTTCGCAAATGGTCAATTGACCATTAGATACATTTTTTTTT

AATGGGATGCGCCATTCGCCATTCAGGCTTTTTTTTT

TTTTTTTTTGATATAAGTATAGCCCGGAATAGGTGTA

TCTGAAACGATAAGTGCCGTCGAGAGGGTTTTTTTTT

TTTTTTTTAGTCTCTGAATTTACCCCAGAATGGAAAGCGCTTTTTTTT

TTTTTTTTGTTGATAATCAGAAAAGCCCCTTCCTGTA

TTTTTTTTGCATAAAGTGTAAAGCCTGGGGTGCCTAATTCCA

TTTTTTTTATCCAATAAATCATACAGGCATGACCCTG

TTTTTTTTGGAAGTTTCATCGAGTAGATTTAGTTAACCTGTTTAGCTATA

TTTTTTTTAAGTTTATTTTGTCACAATCAAATTAGAG

TTTTTTTTCAGGTCGACTCTAGAGCAAGCTTGCATGCCTGTTTTTTTT

TTTTTTTTCAGAGAGATAACCTTTTTAAGAAAAGTAACCGAGG

CAGAACCGCCACCCTTTTTCAGGGATAGCAAGTTTCGTCACCTTTTTTTT

TTTTTTTTAAAGTTACCAGAAGGAAAGCAGATAGCCGAACTTTTTTTT

TCAATTCTACTAATAGTAGTAGCATTAACTTTTTTTT

TTTTTTTTAACGATTTTTTGTTTAACGTCTGAACACC

ACAGTTGATAAATATGCAACTAAAGTACGGTGTCTTTTTTTTT

Design and sequences of CDL

caDNAno design of CDL

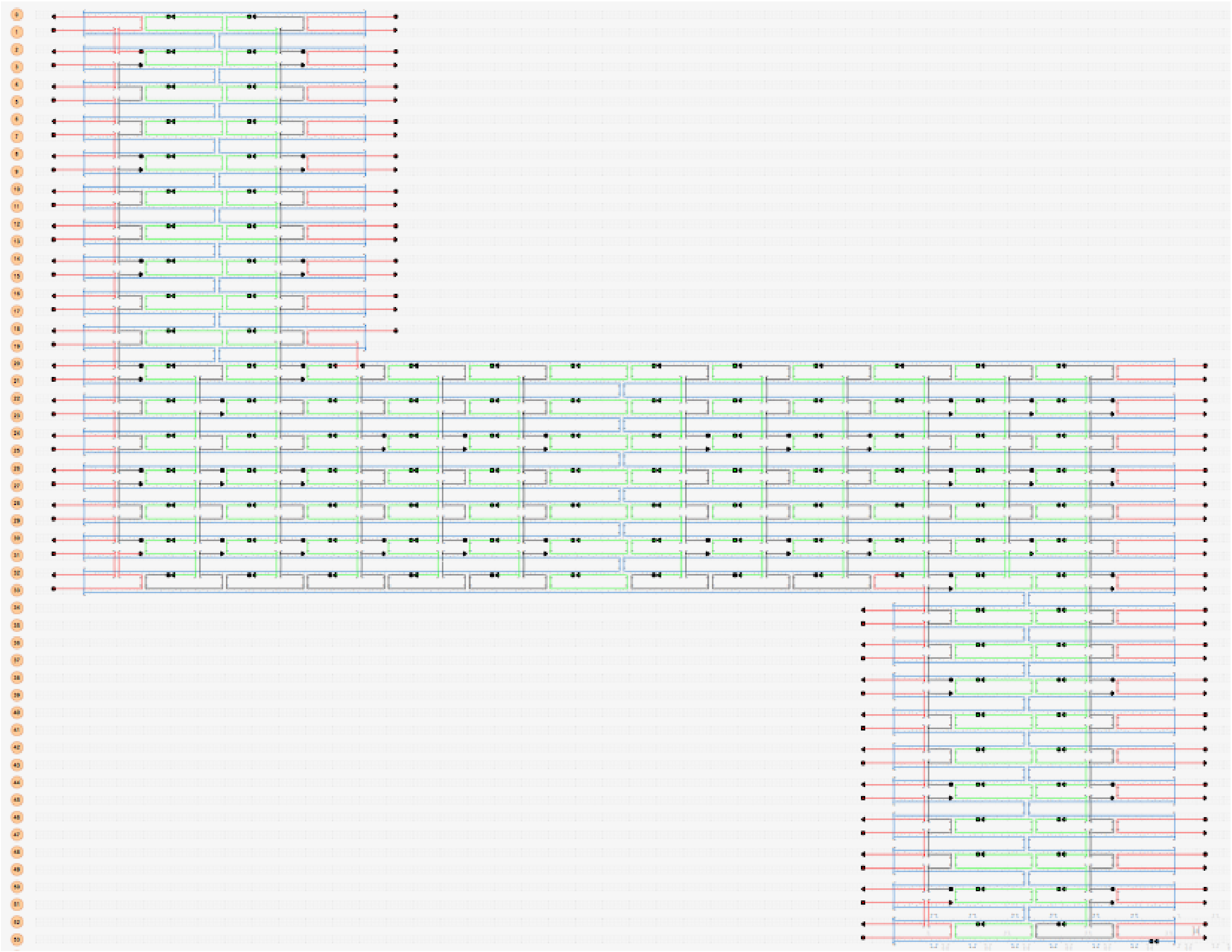

Staple strand sequences (5’ → 3’) for CDL (in total 220 strands)

Core staples pool #1 (green, 114 staples):

AAGCGCATGAGGCAGGTCAGACCAGCAT
TTTTTTCAGGAGCCTTTAATTGTATCGTGTTGCGAATAATAA
TTTTAACCTGAATTTACCGTTGAATGGA
CTAAACAAAGGAACAACTAAAGGAATCTGGTATGGGATTTTG
AGATAGGCACTATTAAAGAACGTGGACTGAAAGAATAGCCCG
CGTTGTACCTGAGAAGTGTTTGGAACGG
CAGGCGAAAAATCCCTTATAAATCAAATGCGGTTTGCCCCAG
AATATCCTACTTCTTTGATTAAATTAAC

ACAGCTGGCAGCAAGCGGTCCACGCTAAAGGTGAGACGGGCA
CGTCTGACAATATTACCGCCATGCTGGT
AACGCGCGTGGTTTTTCTTTTCACCAAAAAAATGAATCGGCC
GATAGAAGATTATTTACATTGGCTCAAT
ATTGCGTCTGTCGTGCCAGCTGCATTTGCCTAACTCACATTA
GCGAACTTCTGACCTGAAAGCCAACAGA
ACACAACGGGTGCCTAATGAGTGAGCCTGAGCTCACAATTCC
GTCAGTAGCCCTAAAACATCGTTAATGC
TACCGAGCTGTGTGAAATTGTTATCCTATTAGGATCCCCGGG
TCTGAAATCAGTGCCTTGAGTTAATAAG
TTAGCGTTTAGTAAATGAATTTTCTGGGCACAGCCCTCATAG
AACCTCACACCGCCTGCAACATGAGGCG
CACGACGGCCTGCAGGTCGACTCTAGAAAAGGTTTTCCCAGT
TAAAATATCAAACCCTCAATCCTTGCTG
TTACGCCTTAAGTTGGGTAACGCCAGTATCGCCTCTTCGCTA
AGACTTTTAGGAGCACTAACAGGTTATC
GGCAAAGGGAAGGGCGATCGGTGCGGTTACTGCCGGAAACCA
ATCATTTACAATTCGACAACTAAGTATT
ACCACCATTTCCGGCACCGCTTCTGGAATGCGGAAACAGTAA
CCGGTTGTGGGATAGGTCACGTTGGTGTTGATCATATGTACC
TTTAAATGCCTGAGAGTCTGGGATTGTA
ATGTGTATACAAAGGCTATCAAATTGTA
AGGGTGAGGTAGCTATTTTTGGTTAAAA
AGTCAAATGATAAATTAATGCTTGTTAA
ATCGCGCCAGTACCTTTTACATAACATT
CATTTCACGTCAGATGAATATCAAAGAA
AGAAGATTGCGTAGATTTTCACGGAATT
CAAGAAAGTAAAACAGAAATAGATTATC
ATTTAACCCATATCAAAATTACATCAAT
TACCTTTTAATGGAAGGGTTATTGGATT
TATTTCATCGTAAAACTAGCATGTCAAATTGGGAGAAGCCTT
ATTTTTAAAGAGAATCGATGAGAAAAGC
TTTGACCCCTCAGAGCATAAACATATAT
GCAAATGTAGCAAAATTAAGCCTGAGTA
TAGCTATCATACAGGCAAGGCATTCAAA
CGCGAGCTAGCATTAACATCCCGGAGAC
TTTTAACCTTAGATTAAGACGTTACAAA
GGGTTATTCCTTGAAAACATAAATTATT
ATGCTGATTAATTAATTTTCCGAGCAAA
CAAGACATGCTTCTGTAAATCCAAACAT
ACTTTTTATATATGTGAGTGATAATTAC
TTAATTTATGGAAACAGTACATTTGAAT
ATATAACGACCCTGTAATACTTTTGCTACAAAGTTTCATTCC

TCTGCGACGGTTGTACCAAAAGATAAAA
TCAAAAATGCTGAATATAATGGATTTAG
CCGAAAGGCGGATGGCTTAGAACATTTC
GTTTTAATTTTGATAAGAGGTACCTGTT
CGAACCAGAGAGTACCTTTAATTTGGGG
CGCCATATCATATGCGTTATAACTACCT
CATGTAACTAGAAAAAGCCTGTTAGGTT
TTTTCGATAAACACCGGAATCTATGTAA
AATATAAAAATAAGGCGTTAACCAATCG
GTAAAGTTTGAAATACCGACCCGAGAAA
GACGACATTCTGACCTAAATTATTTTAG
TCTTTACACTAAAGTACGGTGTCTGGTCATTCAAAAATCAGG
TCAGAAGTCAACATGTTTTAATCCCAAT
AAAGGAAAATATTCATTGAATGATTGCA
AAGAGCACAATACTGCGGAATAGGAAGC
CTCGTTTATGTTTAGACTGGAATATCGC
AAAACCAGCCAGAGGGGGTAATTCAAAG
GAACAAGTCAATAATCGGCTGTGAGAAT
TTTCATCTTACGAGCATGTAGACGCCAA
CGCGCCCAAAATAATATCCCAAGAGGCA
CAGATATATAGATAAGTCCTGATAAGAG
GTATTCTGAACGCGCCTGTTTACAAAAG
TTTTAGCACAACATGTTCAGCTCCAGAC
TTTAGGAAAACGAGAATGACCATAAAAAGACATCAGTTGAGA
TAATGCAAAATGCTTTAAACAATTATAG
TCAACGTAAGAAAAATCTACGAACGCCA
TTCAGTGTATACCAGTCAGGAGCATAGT
TGACGAGCGATTTTAAGAACTCATAACC
GTAGTAAATCATTGTGAATTAGACGATA
ATTTTTTCCAGAGCCTAATTTTCATCGA
ATGAAAACCAACGCTAACGAGTTTTTAT
GAGAGAAACAATTTTATCCTGTCATTAC
GGAAGCGGTTGCTATTTTGCAAGCAAAT
ATTAACTTGAAGCCTTAAATCTTATCCG
AAGTCAGCTCCCGACTTGCGGCGAGGCG
TCAAGAGTATTACAGGTAGAAAGATTACAGGGCTGACCTTCA
ACCGGATAACGAACTAACGGAATTCAAC
CAAAGACACCGAACTGACCAAACCCAAA
CAACCGACAGACGGTCAATCACTGCTCA
GTAAATATTACTTAGCCGGAACTTGCCC
ACAACGGAAATCCGCGACCTGAGAACGA
GGAATACTTTAAGAAAAGTAAAGAAACG
ATGATTAAGCTATCTTACCGAGTCAAAA

GCAGTATAAGAAACAATGAAACTTTACA
AAAATACGTTAAGCCCAATAAAAAACAG
CAACATAGAGATAACCCACAACGGGAGA
AGACACCTAATTGAGCGCTAACTGAACA
TTTTGTCCAGACCAGGCGCATAGGCTGGACGGAATAAGTTTA
ATTCATAAGAGGACAGATGAAGACAAGA
GCGGATAAAAGTATTAAGAGGCTATTAT
ACCGTAAGCCTGTAGCATTCCACAGATGAGAGGAACCCATGT
CAAAATCCGGAAATTATTCATAGGGAAG
AAGAGGCCCCAGCGATTATACCAAGCGAACAACCTAAAACGA
CAGTAGCGTAGCACCATTACCGAGCCAG
TTTCATGATGCCACTACGAAGGCACCCAGAGACTAAAGACTT
TATTAGCGAATCAAGTTTGCCCCGTAAT
CCTCAGCCGGCTACAGAGGCTTTGAGCAGTCGGGATCGTCAC
CACCCTCGCCATCTTTTCATAGCCCCCT
CACGCATGAGTTAAAGGCCGCTTTTGTTAGACAACCATCGCC
TGACAGGCCGCCACCCTCAGAGAGCCGC
TGCTTTCAGTTGCGCCGACAATGACAAAAGGGTTTATCAGCT
CGCCACCCCGTCGAGAGGGTTGTACCAG
CCTCAGACAGGGATAGCAAGCCCAATTGCTCAGAACCGCCAC

Core staples pool #2 (black, 50 staples):

GCTTAATTCTTTCCACCGCACGCCAGTT
TTTAGGCTCCTAATGTAGGAAAATCTTA
AATTTCATAAATCACAAATATTAATGGTAATTCTGTAATGCA
GATGAAAGTCGCTATGCAAATATAAGAAGCCAGTAAACAAGA
ACGTTGAGAATAGAACTTTCACCAGACGAACGATCCTACAAC
GTTTGAGTCGGGAGTACCAAGCTGAGAACTGAGAGCAAATTC
ATAATCCGCATCTGCCAGTTTGAGGGGAGGCAATTTTTGCAC
TTCGCATGTAGCCAGCTTTCATCAACATATATTTTAGAGATC
AAACACCCTCCATGTTGCGAAACAAAGT
TACGCCACCAACGTCAAAGGGCGAAAAATTAGACATTATAAT
AGCGAAATTGCAGGAACCGATTACCGATGAGGTGACTCCAAA
TTGAGGGTAAAGGTGGAATTAATTAGCAAGCAGCATTTAGCG
TGCGCTCAAGCCTGATACGAGCTGTTTCCTCGAATCTTGCAT
ATCAGCTTAATTCGCGTCTGGCCTTCCTTAAATTTCGGAGAG
TTTAACAAAACCAACAAGCCGCGTCTTT
TTGTAAAAAGGCGAAGCTGGCACTGTTGCGCCATTAGCCAGC
ACAAAATATAACCTAAGAACGGTGTGATAGTACCGATCAACA
TAAGCAACCGTCGGATTCTCCGTGGGAAACAGGAAAGCAAAC
AACGTTATAAATGTGAGCGAGTAACAACATATTTAGGTCATT
TCACCATATAGTAGTGAAAAGAGGATTA

GACCGGAAAGTTTTAAATAGCAACTTTAATTGGGCTGTGTCG
ACTTCAATAGCGTCACACTATGGCTCATAATAAGGCGAGGCG
TCACGCAGTAATAATCGGCCTGCCATTGTTTTGACGCAGATT
AGATGATCGACGACAGTATCGGCCTCAGTATTCCTAAGAAAT
GCATCACAATATCTTGAGGAAACTAATAGATTTAGCGTATTA
GAACCCTGCTAAATACGAGTACTGTAGCCAAAGCGCCCCCTC
AATAGCACCCAGCTTAACATATAAGAGCGTTAGCAAACGTAG
CCAAATAGCAGATAAACGCAATAATAAC
GAAAGGCAATAAATATTTTCATTGCTCC
AGAAGGCAAGATTACATTAGAGAATTGAATACATAAAGGTGG
TAGCAGCTAGCAATAGACTCCTTATTAC
TTCGAGCTAGTAAAACCAGACCCTTATG
GAAGGAGGGTTTAAATTACCTCTTAGAAATAACTAATAATTA
TTGCTCAGATATAAAGGAGGTTTAGTAC
AAAGCCACCAGTAAGTACTGGAACAGTGTCGGAACCTGAGAC
CCCAAAACAAACGGCGGATTGACCGTAAATAATCAACGGTAA
ATACCACACAACATTAATCTTCGGTGTAACAATCAATAGAAA
AGATTTGTTTGACCAAAAGAAATACGTAAGGAAGTGTAGCAA
AAATCCTGAGAGTTATTGCCCCGCCAGGGGGGAGAGGGAAAC
GATACATTTAATAAATTCATTCTTTGAATGGTTTACCAGCGC
TTACGAGCGTTGGGAACAAAGTAAGGGAAAAAGGG
ACGCAAGACATTATAGTTGATATATGCACCTGACTGTTCAGA
GTTTAACAGCCCTTCCAAAAGAACTGGC
AAGAACGGAGGTTTGAACACCTATCAGATAAAAGAAACGCAA
GGTAAAGAAAGAATGTCAATACATTTTT
AGAGGCGGCGATAGCTCCGGCTTTAGTA
GCAATGCAATAAAGATTAGATGCTTAATGATTAAGCGTCATA
TTCTGGCGTAAGAATTAGTCTCCATTAAACAGAGGGTGCCAC
ATACTTCAGATGGGCGCATCGTAACCGTTGATTGTGAACCTA
GGTCATAATCAAAATCCCTCAGCCACCAGCCGCCGCGATTGG

Sidestrands with poly-T overhangs (black, 56 staples):

TTTTTTTTATGCCCCCTGCCTATTCCCGTATAAACAGTTATTTTTTTT
TTTTTTTTGTTATTAATTTTAAAAAATCCTTTGCCCGAACTTTTTTTT
TTTTTTTTAGATAATACATTTGAGGATTAGAGCCGTCAATTTTTTTTT
TTTTTTTTATTCAACCGTAACCAATATTTTTTTTT
TTTTTTTTGGAACGAGGTTCCATTAATTTTTTTTT
TTTTTTTTCAGAACCACCACCAGACCCTCAGAGCCGCCACTTTTTTTT
TTTTTTTTGGAACGCCATCAAAAACATTTTTTTCTAGC
TTTTTTTTGTGTATCACCGTACTCGTATAGCCCGGAATAGTTTTTTTT
TTTTTTTTTATTTTTGAATGGCTATACGTGGCACAGACAATTTTTTTT
TTTTTTTTAGAAGGAAACCGAGGAGCCGAACAAAGTTACCTTTTTTTT

TTTTTTTTCAACAGGTCGTGGCATCATTTTTTTT

TTTTTTTTTTTAATTTCGAGAGGCTTTTTTTTTT

TTTTTTTTTTTCATCGGCATTTTCTCAGACTGTAGCGCGTTTTTTTTT

TTTTTTTTCACCACCGGAACCGCCTCACCGGAACCAGAGCTTTTTTTT

TTTTTTTTATGTGCTGCACGACGGCCTTTTTTTT

TTTTTTTTGGGTATTAAACCAAGTTTATCATTCCAAGAACTTTTTTTT

TTTTTTTTTATTATTTATCCCAATACAAAATAAACAGCCATTTTTTTT

TTTTTTTTCAGTACAAATAAAGTTTTTTTTTTTT

TTTTTTTTTGGTTCCGAGTTGTTCCATTTTTTTT

TTTTTTTTAGTAATAAAAGGGACACACCAGTCACACGACCTTTTTTTT

TTTTTTTTTAAAAGAGTCTGTCCACAGTGAGGCCACCGAGTTTTTTTT

TTTTTTTTGCCTGATTGCTTTGAAAAACAATAACGGATTCTTTTTTTT

TTTTTTTTAACAGTTGAAAGGAATGGTCAGTTGGCAAATCTTTTTTTT

TTTTTTTTTATCAAAATCATAGGTGAGTCAATAGTGAATTTTTTTTTT

TTTTTTTTTAGGATTAGCGGGGTTTCCTCAAGAGAAGGATTTTTTTTT

TTTTTTTTGCCGATTAAAGGGATTCCGTCTATCACAGGAGTTTTTTTT

TTTTTTTTACCAGCAGAAGATAAAAAATACCGAACGAACCTTTTTTTT

TTTTTTTTGTCGTCTTTACAGTTTCATTTTTTTT

TTTTTTTTCGTATTGGGTTCACCGCCTTTTTTTT

TTTTTTTTAACGCTCAACAGTAGGTTACCAGTATAAAGCCTTTTTTTT

TTTTTTTTTTTCCAGTCGGCGGTTTGTTTTTTTT

TTTTTTTTGTTTGGAACAAGAGTCGTTGAGTAATCGGC

ATCATCAGAAGATCGCACTCCCGCCATTCATTTTTTTT

TTTTTTTTCCACCACCCTTTCGTCACTTTTTTTT

TTTTTTTTACGGGTAAATACACTAAATTTTTTTT

TTTTTTTTCAATGAAACCATCGATAGGCCGGAAACGTCACTTTTTTTT

TTTTTTTTTGGCCCTGAGTTTGATGGTTTTTTTT

TTTTTTTTAGAAGAACTCAAACTACATCACTTGCCTGAGTTTTTTTTT

CACTGAGTCATTTTACCGCCACCCTCAGAGTTTTTTTT

TTTTTTTTGCGGAGTGAAAATCTCCATTTTTTTT

TTTTTTTTAAATGAAAAATCTAAAGCTGAGAGCCAGCAGCTTTTTTTT

TTTTTTTTCTGATAAATTTGAGATGGTTTTTTTT

TTTTTTTTTTGATGATACAGGAGTGCGTCATACATGGCTTTTTTTTTT

TTTTTTTTTTGCAAAAGAGCAAACTCTTTTTTTT

TTTTTTTTCGCTGAGGCGACAGCATCTTTTTTTT

TTTTTTTTCATGGAAATACCTACACAACAGGAAAAACGCTTTTTTTTT

TTTTTTTTTGGTCATAGCCGGAAGCATTTTTTTT

TTTTTTTTGGCTGCGCAGAAAGGGGGTTTTTTTT

TTTTTTTTAAAAAAAGGATTTCTTAATTTTTTTT

TTTTTTTTACACTCATCTATCATCGCTTTTTTTT

TTTTTTTTTAAAGTGTAACTGCCCGCTTTTTTTT

TTTTTTTTCGACTTGAGCCATTTGGAATTATCACCGTCCGACATT

TTTTTTTTATTCTACTACAATATGATTTTTTTTT

TTTTTTTTAGTGCCAAGTCGTAATCATTTTTTTT
TTTTTTTTCAAATAAATCCTCATTCCTTGATATTCACAAATTTTTTTT
TTTTTTTTACAGCTTGAATATTCGGTTTTTTTTT

Extended staples for biasing the orientation of CDL (these strands replace core strands pool #1) (114 staples):

TTTTTTTTTTTTTTTTTTTTAAGCGCATGAGGCAGGTCAGACCAGCAT
TTTTTTTTTTTTTTTTTTTTTTTTTTCAGGAGCCTTTAATTGTATCGTGTTGCGAATAATAA
TTTTTTTTTTTTTTTTTTTTTTTTAACCTGAATTTACCGTTGAATGGA
TTTTTTTTTTTTTTTTTTTTCTAAACAAAGGAACAACTAAAGGAATCTGGTATGGGATTTTG
TTTTTTTTTTTTTTTTTTTTAGATAGGCACTATTAAAGAACGTGGACTGAAAGAATAGCCCG
TTTTTTTTTTTTTTTTTTTTCGTTGTACCTGAGAAGTGTTTGGAACGG
TTTTTTTTTTTTTTTTTTTTCAGGCGAAAAATCCCTTATAAATCAAATGCGGTTTGCCCCAG
TTTTTTTTTTTTTTTTTTTTAATATCCTACTTCTTTGATTAAATTAAC
TTTTTTTTTTTTTTTTTTTTACAGCTGGCAGCAAGCGGTCCACGCTAAAGGTGAGACGGGCA
TTTTTTTTTTTTTTTTTTTTCGTCTGACAATATTACCGCCATGCTGGT
TTTTTTTTTTTTTTTTTTTTAACGCGCGTGGTTTTTCTTTTCACCAAAAAAATGAATCGGCC
TTTTTTTTTTTTTTTTTTTTGATAGAAGATTATTTACATTGGCTCAAT
TTTTTTTTTTTTTTTTTTTTATTGCGTCTGTCGTGCCAGCTGCATTTGCCTAACTCACATTA
TTTTTTTTTTTTTTTTTTTTGCGAACTTCTGACCTGAAAGCCAACAGA
TTTTTTTTTTTTTTTTTTTTACACAACGGGTGCCTAATGAGTGAGCCTGAGCTCACAATTCC
TTTTTTTTTTTTTTTTTTTTGTCAGTAGCCCTAAAACATCGTTAATGC
TTTTTTTTTTTTTTTTTTTTTACCGAGCTGTGTGAAATTGTTATCCTATTAGGATCCCCGGG
TTTTTTTTTTTTTTTTTTTTTCTGAAATCAGTGCCTTGAGTTAATAAG
TTTTTTTTTTTTTTTTTTTTTTAGCGTTTAGTAAATGAATTTTCTGGGCACAGCCCTCATAG
TTTTTTTTTTTTTTTTTTTTAACCTCACACCGCCTGCAACATGAGGCG
TTTTTTTTTTTTTTTTTTTTCACGACGGCCTGCAGGTCGACTCTAGAAAAGGTTTTCCCAGT
TTTTTTTTTTTTTTTTTTTTTAAAATATCAAACCCTCAATCCTTGCTG
TTTTTTTTTTTTTTTTTTTTTTACGCCTTAAGTTGGGTAACGCCAGTATCGCCTCTTCGCTA
TTTTTTTTTTTTTTTTTTTTAGACTTTTAGGAGCACTAACAGGTTATC
TTTTTTTTTTTTTTTTTTTTGGCAAAGGGAAGGGCGATCGGTGCGGTTACTGCCGGAAACCA
TTTTTTTTTTTTTTTTTTTTATCATTTACAATTCGACAACTAAGTATT
TTTTTTTTTTTTTTTTTTTTACCACCATTTCCGGCACCGCTTCTGGAATGCGGAAACAGTAA
TTTTTTTTTTTTTTTTTTTTCCGGTTGTGGGATAGGTCACGTTGGTGTTGATCATATGTACC
TTTTTTTTTTTTTTTTTTTTTTTAAATGCCTGAGAGTCTGGGATTGTA
TTTTTTTTTTTTTTTTTTTTATGTGTATACAAAGGCTATCAAATTGTA
TTTTTTTTTTTTTTTTTTTTAGGGTGAGGTAGCTATTTTTGGTTAAAA
TTTTTTTTTTTTTTTTTTTTAGTCAAATGATAAATTAATGCTTGTTAA
TTTTTTTTTTTTTTTTTTTTATCGCGCCAGTACCTTTTACATAACATT
TTTTTTTTTTTTTTTTTTTTCATTTCACGTCAGATGAATATCAAAGAA
TTTTTTTTTTTTTTTTTTTTAGAAGATTGCGTAGATTTTCACGGAATT

TTTTTTTTTTTTTTTTTTTTCAAGAAAGTAAAACAGAAATAGATTATC

TTTTTTTTTTTTTTTTTTTTATTTAACCCATATCAAAATTACATCAAT

TTTTTTTTTTTTTTTTTTTTTACCTTTTAATGGAAGGGTTATTGGATT

TTTTTTTTTTTTTTTTTTTTTATTTCATCGTAAAACTAGCATGTCAAATTGGGAGAAGCCTT

TTTTTTTTTTTTTTTTTTTTATTTTTAAAGAGAATCGATGAGAAAAGC

TTTTTTTTTTTTTTTTTTTTTTTGACCCCTCAGAGCATAAACATATAT

TTTTTTTTTTTTTTTTTTTTGCAAATGTAGCAAAATTAAGCCTGAGTA

TTTTTTTTTTTTTTTTTTTTTAGCTATCATACAGGCAAGGCATTCAAA

TTTTTTTTTTTTTTTTTTTTCGCGAGCTAGCATTAACATCCCGGAGAC

TTTTTTTTTTTTTTTTTTTTTTTTAACCTTAGATTAAGACGTTACAAA

TTTTTTTTTTTTTTTTTTTTGGGTTATTCCTTGAAAACATAAATTATT

TTTTTTTTTTTTTTTTTTTTATGCTGATTAATTAATTTTCCGAGCAAA

TTTTTTTTTTTTTTTTTTTTCAAGACATGCTTCTGTAAATCCAAACAT

TTTTTTTTTTTTTTTTTTTTACTTTTTATATATGTGAGTGATAATTAC

TTTTTTTTTTTTTTTTTTTTTTAATTTATGGAAACAGTACATTTGAAT

TTTTTTTTTTTTTTTTTTTTATATAACGACCCTGTAATACTTTTGCTACAAAGTTTCATTCC

TTTTTTTTTTTTTTTTTTTTTCTGCGACGGTTGTACCAAAAGATAAAA

TTTTTTTTTTTTTTTTTTTTTCAAAAATGCTGAATATAATGGATTTAG

TTTTTTTTTTTTTTTTTTTTCCGAAAGGCGGATGGCTTAGAACATTTC

TTTTTTTTTTTTTTTTTTTTGTTTTAATTTTGATAAGAGGTACCTGTT

TTTTTTTTTTTTTTTTTTTTCGAACCAGAGAGTACCTTTAATTTGGGG

TTTTTTTTTTTTTTTTTTTTCGCCATATCATATGCGTTATAACTACCT

TTTTTTTTTTTTTTTTTTTTCATGTAACTAGAAAAAGCCTGTTAGGTT

TTTTTTTTTTTTTTTTTTTTTTTTCGATAAACACCGGAATCTATGTAA

TTTTTTTTTTTTTTTTTTTTAATATAAAAATAAGGCGTTAACCAATCG

TTTTTTTTTTTTTTTTTTTTGTAAAGTTTGAAATACCGACCCGAGAAA

TTTTTTTTTTTTTTTTTTTTGACGACATTCTGACCTAAATTATTTTAG

TTTTTTTTTTTTTTTTTTTTTCTTTACACTAAAGTACGGTGTCTGGTCATTCAAAAATCAGG

TTTTTTTTTTTTTTTTTTTTTCAGAAGTCAACATGTTTTAATCCCAAT

TTTTTTTTTTTTTTTTTTTTAAAGGAAAATATTCATTGAATGATTGCA

TTTTTTTTTTTTTTTTTTTTAAGAGCACAATACTGCGGAATAGGAAGC

TTTTTTTTTTTTTTTTTTTTCTCGTTTATGTTTAGACTGGAATATCGC

TTTTTTTTTTTTTTTTTTTTAAAACCAGCCAGAGGGGGTAATTCAAAG

TTTTTTTTTTTTTTTTTTTTGAACAAGTCAATAATCGGCTGTGAGAAT

TTTTTTTTTTTTTTTTTTTTTTTCATCTTACGAGCATGTAGACGCCAA

TTTTTTTTTTTTTTTTTTTTCGCGCCCAAAATAATATCCCAAGAGGCA

TTTTTTTTTTTTTTTTTTTTCAGATATATAGATAAGTCCTGATAAGAG

TTTTTTTTTTTTTTTTTTTTGTATTCTGAACGCGCCTGTTTACAAAAG

TTTTTTTTTTTTTTTTTTTTTTTTAGCACAACATGTTCAGCTCCAGAC

TTTTTTTTTTTTTTTTTTTTTTTAGGAAAACGAGAATGACCATAAAAAGACATCAGTTGAGA

TTTTTTTTTTTTTTTTTTTTTAATGCAAAATGCTTTAAACAATTATAG

TTTTTTTTTTTTTTTTTTTTTCAACGTAAGAAAAATCTACGAACGCCA

TTTTTTTTTTTTTTTTTTTTTTCAGTGTATACCAGTCAGGAGCATAGT

TTTTTTTTTTTTTTTTTTTTTGACGAGCGATTTTAAGAACTCATAACC
TTTTTTTTTTTTTTTTTTTTGTAGTAAATCATTGTGAATTAGACGATA
TTTTTTTTTTTTTTTTTTTTATTTTTTCCAGAGCCTAATTTTCATCGA
TTTTTTTTTTTTTTTTTTTTATGAAAACCAACGCTAACGAGTTTTTAT
TTTTTTTTTTTTTTTTTTTTGAGAGAAACAATTTTATCCTGTCATTAC
TTTTTTTTTTTTTTTTTTTTGGAAGCGGTTGCTATTTTGCAAGCAAAT
TTTTTTTTTTTTTTTTTTTTATTAACTTGAAGCCTTAAATCTTATCCG
TTTTTTTTTTTTTTTTTTTTAAGTCAGCTCCCGACTTGCGGCGAGGCG
TTTTTTTTTTTTTTTTTTTTTCAAGAGTATTACAGGTAGAAAGATTACAGGGCTGACCTTCA
TTTTTTTTTTTTTTTTTTTTACCGGATAACGAACTAACGGAATTCAAC
TTTTTTTTTTTTTTTTTTTTCAAAGACACCGAACTGACCAAACCCAAA
TTTTTTTTTTTTTTTTTTTTCAACCGACAGACGGTCAATCACTGCTCA
TTTTTTTTTTTTTTTTTTTTGTAAATATTACTTAGCCGGAACTTGCCC
TTTTTTTTTTTTTTTTTTTTACAACGGAAATCCGCGACCTGAGAACGA
TTTTTTTTTTTTTTTTTTTTGGAATACTTTAAGAAAAGTAAAGAAACG
TTTTTTTTTTTTTTTTTTTTATGATTAAGCTATCTTACCGAGTCAAAA
TTTTTTTTTTTTTTTTTTTTGCAGTATAAGAAACAATGAAACTTTACA
TTTTTTTTTTTTTTTTTTTTAAAATACGTTAAGCCCAATAAAAAACAG
TTTTTTTTTTTTTTTTTTTTCAACATAGAGATAACCCACAACGGGAGA
TTTTTTTTTTTTTTTTTTTTAGACACCTAATTGAGCGCTAACTGAACA
TTTTTTTTTTTTTTTTTTTTTTTTGTCCAGACCAGGCGCATAGGCTGGACGGAATAAGTTTA
TTTTTTTTTTTTTTTTTTTTATTCATAAGAGGACAGATGAAGACAAGA
TTTTTTTTTTTTTTTTTTTTGCGGATAAAAGTATTAAGAGGCTATTAT
TTTTTTTTTTTTTTTTTTTTACCGTAAGCCTGTAGCATTCCACAGATGAGAGGAACCCATGT
TTTTTTTTTTTTTTTTTTTTCAAAATCCGGAAATTATTCATAGGGAAG
TTTTTTTTTTTTTTTTTTTTAAGAGGCCCCAGCGATTATACCAAGCGAACAACCTAAAACGA
TTTTTTTTTTTTTTTTTTTTCAGTAGCGTAGCACCATTACCGAGCCAG
TTTTTTTTTTTTTTTTTTTTTTTCATGATGCCACTACGAAGGCACCCAGAGACTAAAGACTT
TTTTTTTTTTTTTTTTTTTTTATTAGCGAATCAAGTTTGCCCCGTAAT
TTTTTTTTTTTTTTTTTTTTCCTCAGCCGGCTACAGAGGCTTTGAGCAGTCGGGATCGTCAC
TTTTTTTTTTTTTTTTTTTTCACCCTCGCCATCTTTTCATAGCCCCCT
TTTTTTTTTTTTTTTTTTTTCACGCATGAGTTAAAGGCCGCTTTTGTTAGACAACCATCGCC
TTTTTTTTTTTTTTTTTTTTTGACAGGCCGCCACCCTCAGAGAGCCGC
TTTTTTTTTTTTTTTTTTTTTGCTTTCAGTTGCGCCGACAATGACAAAAGGGTTTATCAGCT
TTTTTTTTTTTTTTTTTTTTCGCCACCCCGTCGAGAGGGTTGTACCAG
TTTTTTTTTTTTTTTTTTTTCCTCAGACAGGGATAGCAAGCCCAATTGCTCAGAACCGCCAC

Additional SEM data set

1) STs on a Si_3N_4 substrate

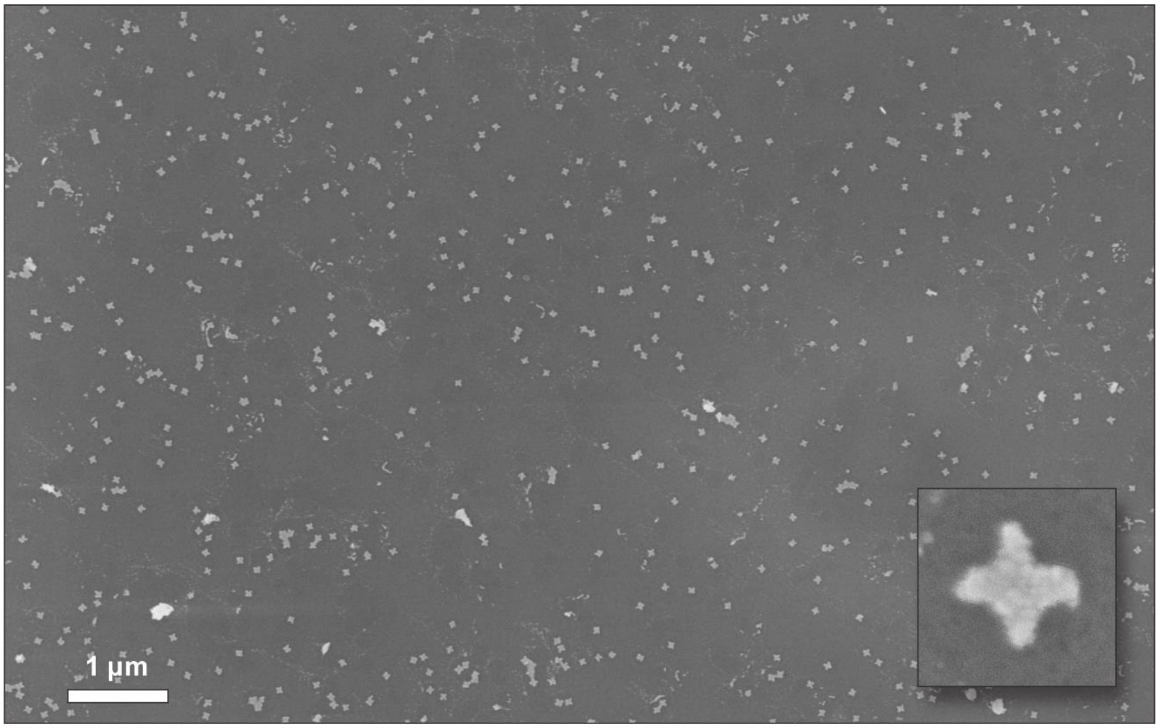

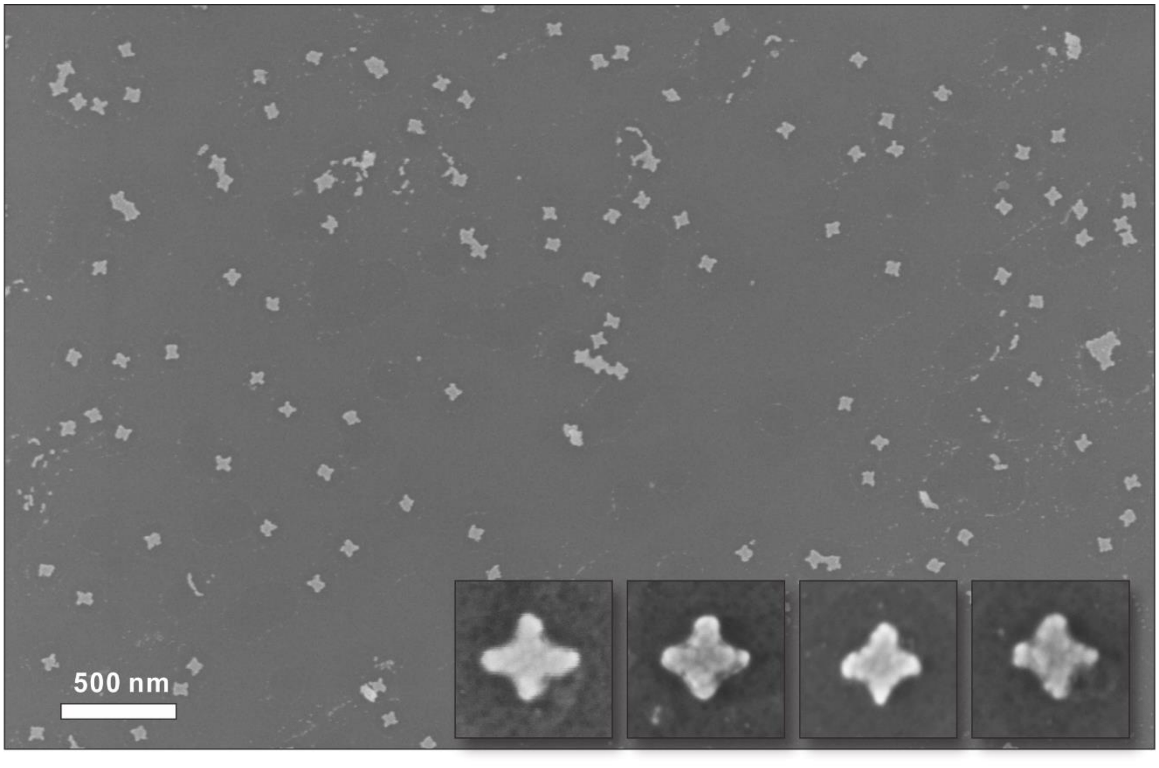

fig. SA1. STs on a Si_3N_4 substrate. The nanostructures are made from gold palladium alloy of 20 nm thickness. Insets show zoomed individual structures. Frame size of the insets is 150 nm × 150 nm.

2) BOs on a Si_3N_4 substrate

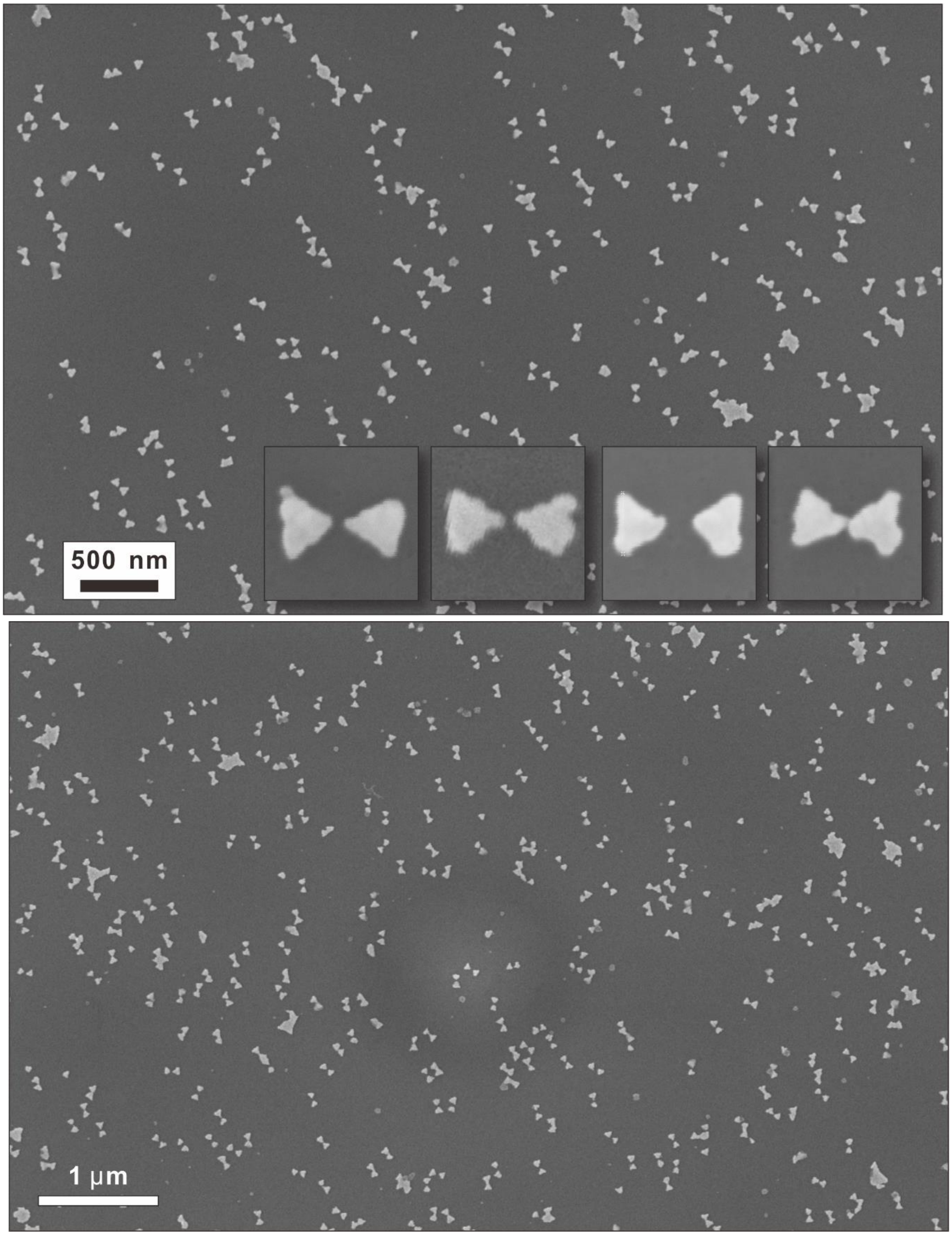

fig. SA2. BOs on a Si_3N_4 substrate. The nanostructures are made from gold of 20 nm thickness. Insets show zoomed individual structures. Frame size of the insets is 150 nm × 150 nm.

BOs on a sapphire substrate

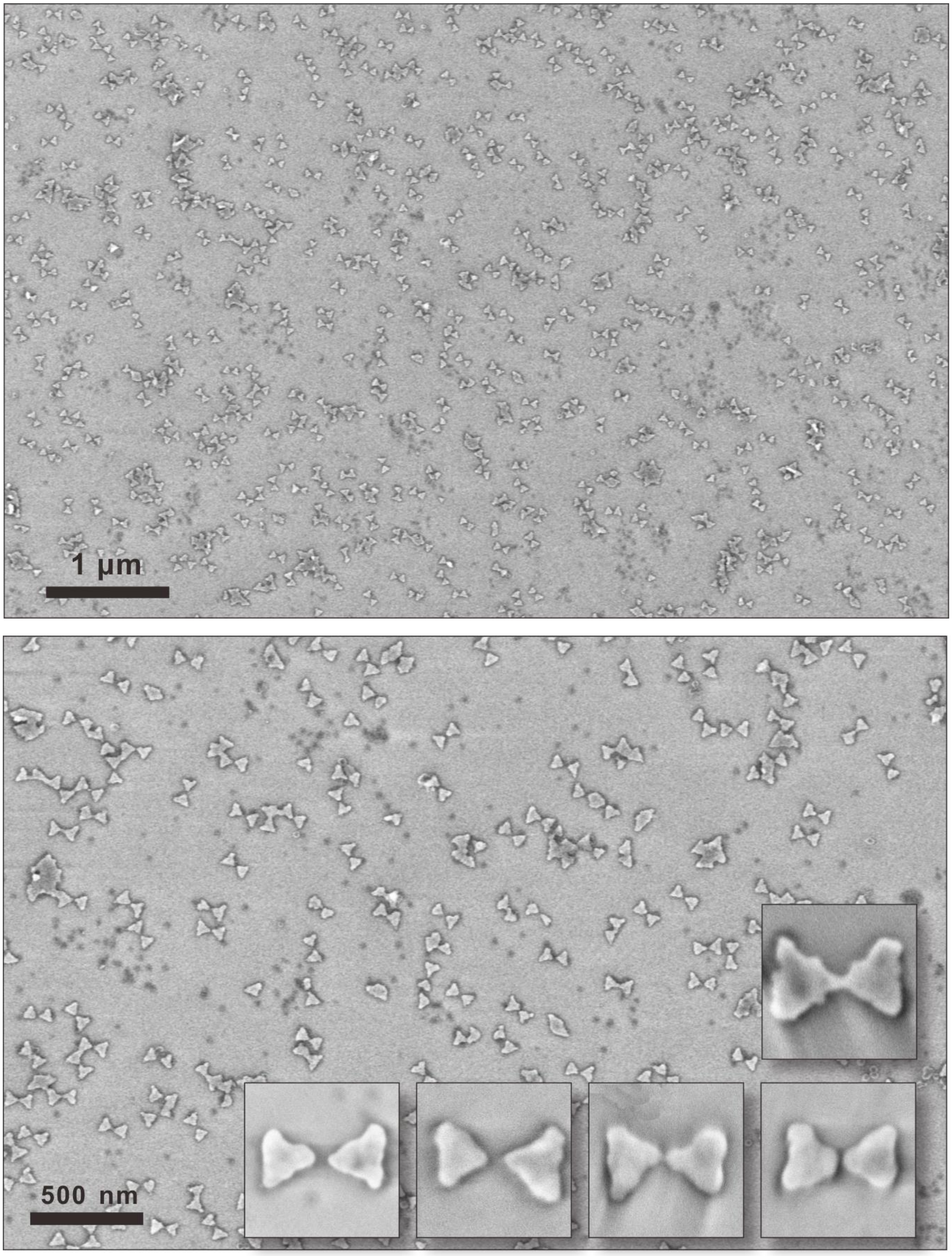

fig. SA3. BOs on a sapphire substrate. The nanostructures are made from gold of 20 nm thickness Insets show zoomed individual structures. Frame size of the insets is 150 nm × 150 nm.

3) CDLs on a sapphire substrate (S-conformation)

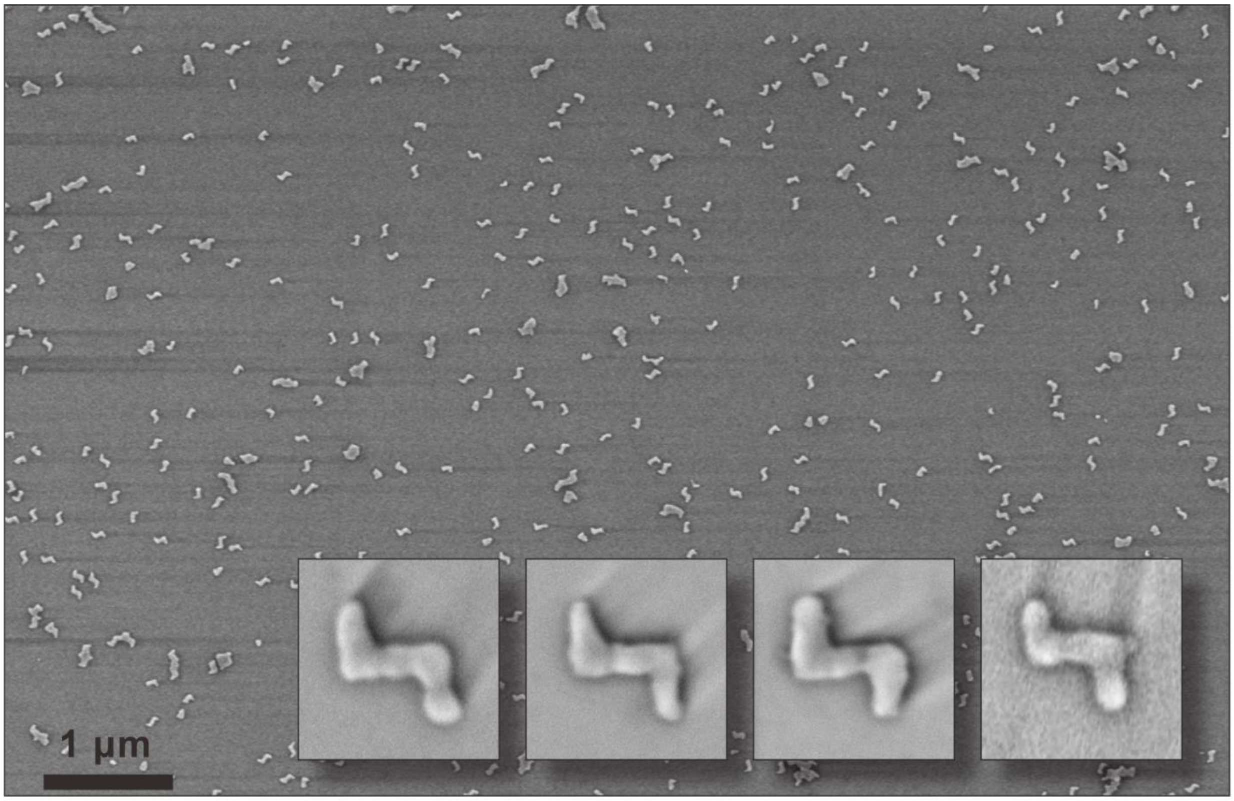

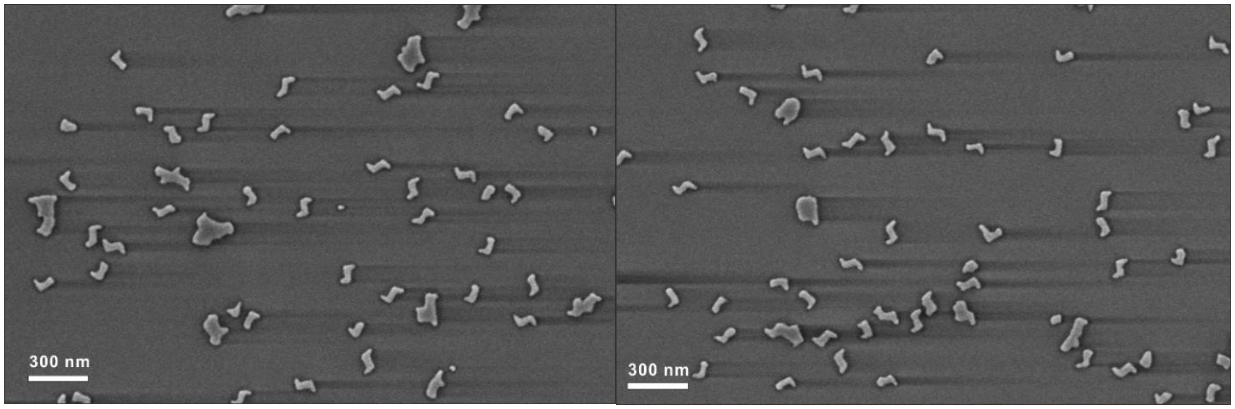

fig. SA4. CDLs on a sapphire substrate. The nanostructures are made from gold of 18 nm thickness and chromium of 2 nm thickness. S-conformation. Insets show zoomed individual structures. Frame size of the insets is 150 nm × 150 nm.

4) CDLs on a sapphire substrate (S- and Z-conformations)

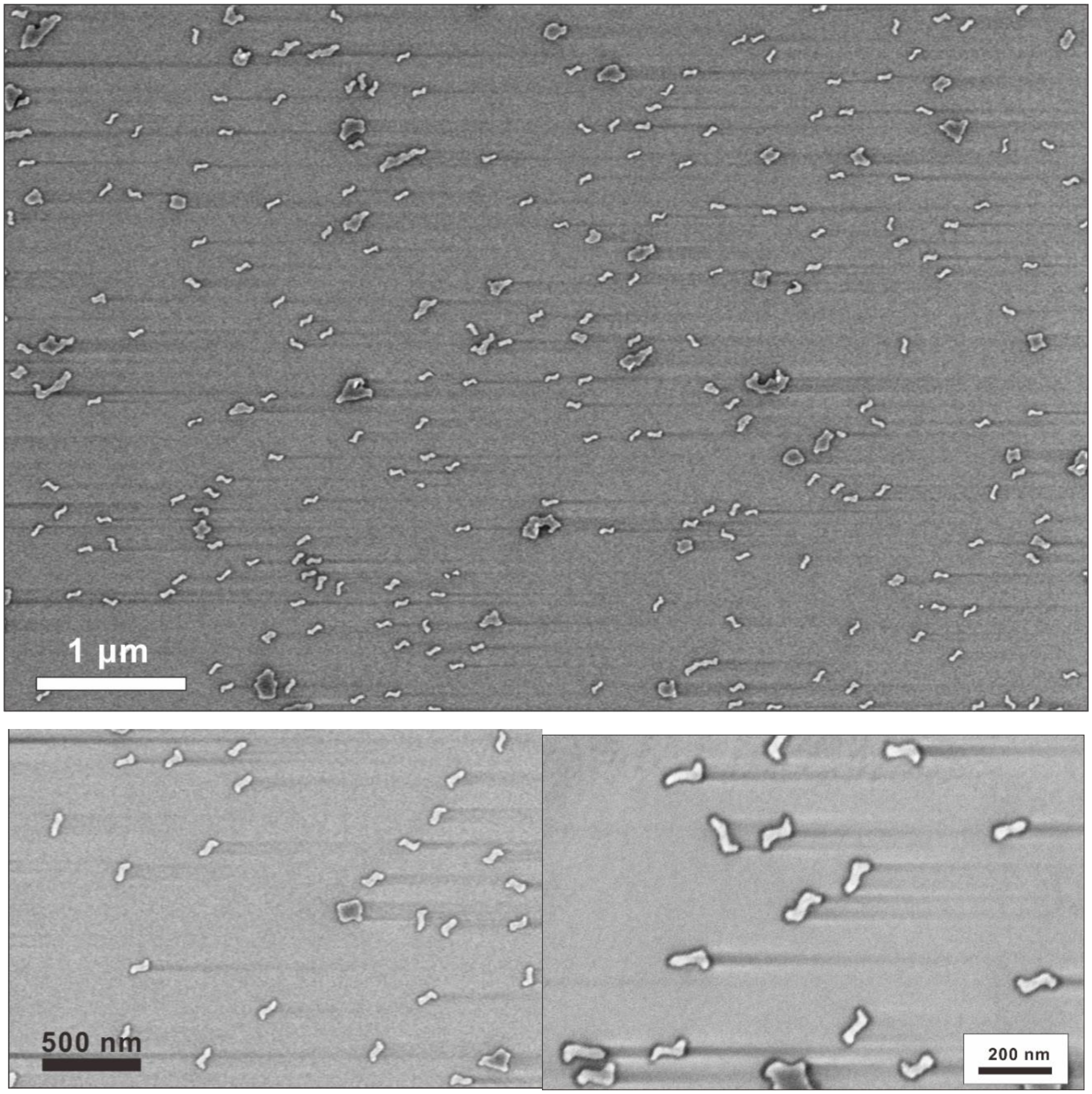

fig. SA5. CDLs on a sapphire substrate. S- and Z-conformations. The nanostructures are made from gold of 18 nm thickness and chromium of 2 nm thickness.

Fabrication yield analysis

The yield of BO metallization was evaluated based on the following criteria: size of the gap, alignment of the triangles, angle between the triangles and the shape of the triangles. BOs were counted as correctly formed if the triangles faced each other with no more than 90° angle between the central axes. If only the BOs with reasonable sized gap (<40 nm) were involved in the calculation of successful BOs, the total fabrication yield was 47 %. If also the BOs without a gap were included, the yield increased to 76 %. The total number of analyzed bowties was 840. Besides the correctly formed BOs and the BOs with missing gaps, one can observe malformed triangles, misaligned triangles and individual triangles without an obvious pair (see Fig. S13). The latter two are mainly caused by the lack of adhesive layer underneath the deposited gold (slight movement of the gold layer during the lift-off process).

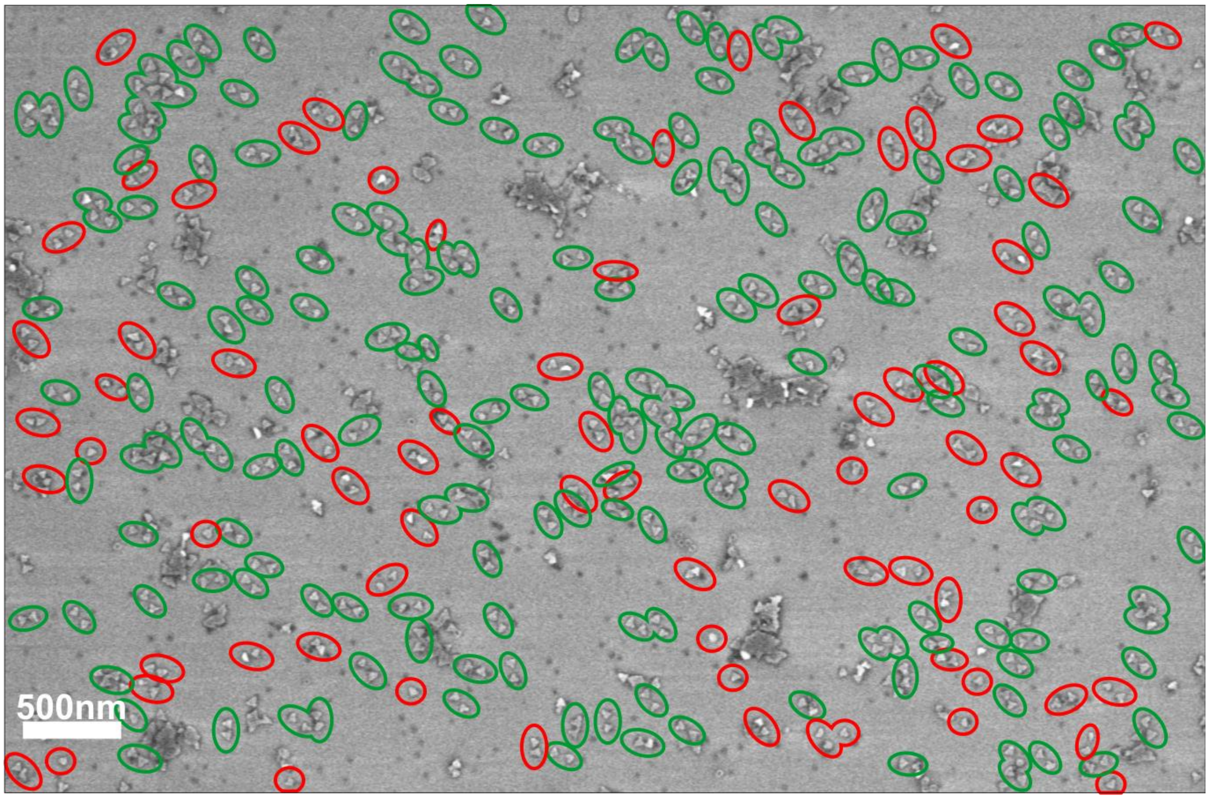

fig. SB1. SEM image of the metallized BOs. The sample are made from 20 nm gold without chromium layer. Correctly and malformed BOs are marked with green and red circles respectively.

The yield of metallization for STs was evaluated based on the criterion that all the four arms of the ST should have been formed correctly (see Fig. S14). In the case where two STs were connected at the ends (but were otherwise well-formed), the structures were counted as correctly formed. The fabrication yield of STs was also 47 %, and the total number of analyzed structures was 758.

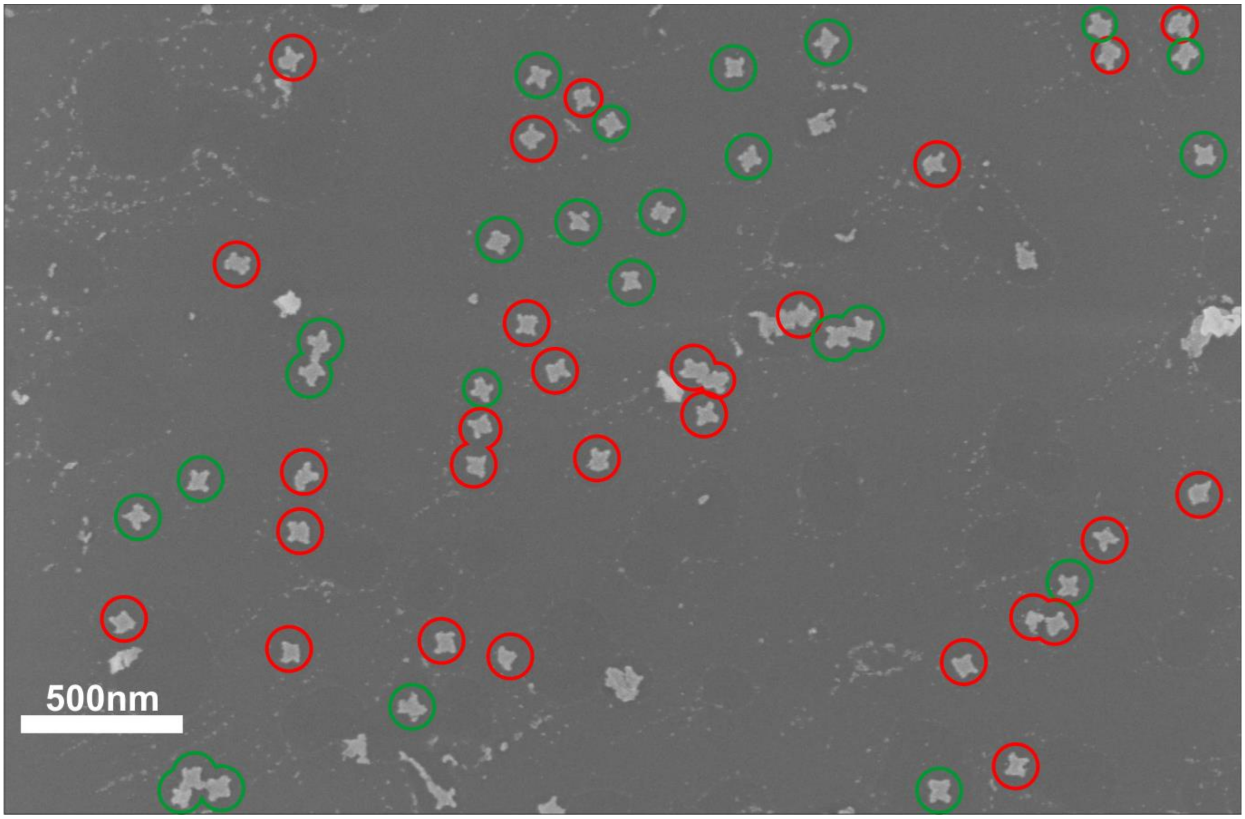

fig. SB2. SEM image of the metallic STs. The sample are made from 20 nm gold without chromium layer. Correctly and malformed STs are marked with green and red circles respectively.

For the CDLs, both the metallization yield (see Figs. S15 and S16) and the selectivity between S- and Z-conformations (see Figs. S17 and S18) were analyzed. For calculating the fabrication yield, both S- and Z-conformations were counted as correctly formed if both arms of the structures were distinguishable and well-formed. The fabrication yield for the sample with the staple extensions was again 47 %, whereas the yield for the sample without the staple strand extensions was 40 %. The main reason for the relatively low yield is related to the origami deposition procedure. It was observed that the flexible arms of the CDL tend to roll up or twist during the deposition resulting in undesired metallic shapes.

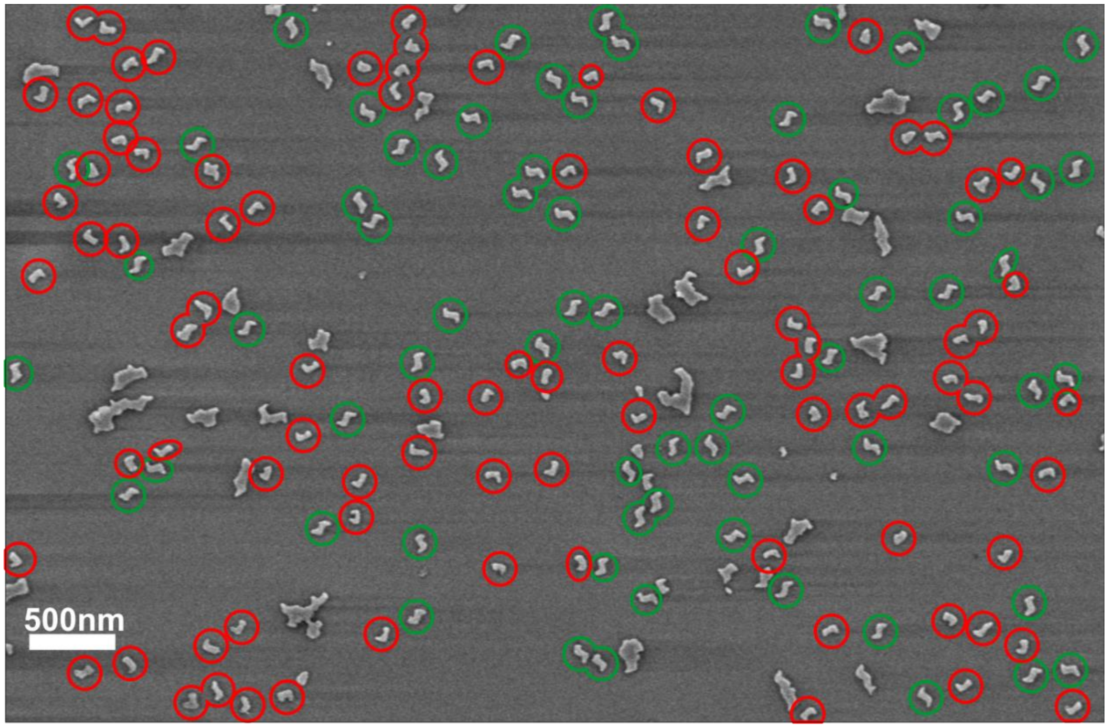

fig. SB3. SEM image of the metallic CDLs (CDL-origamis equipped with staple extensions). The sample are made from 18 nm gold with 2 nm chromium layer. Correctly and malformed CDLs are marked with green and red circles respectively.

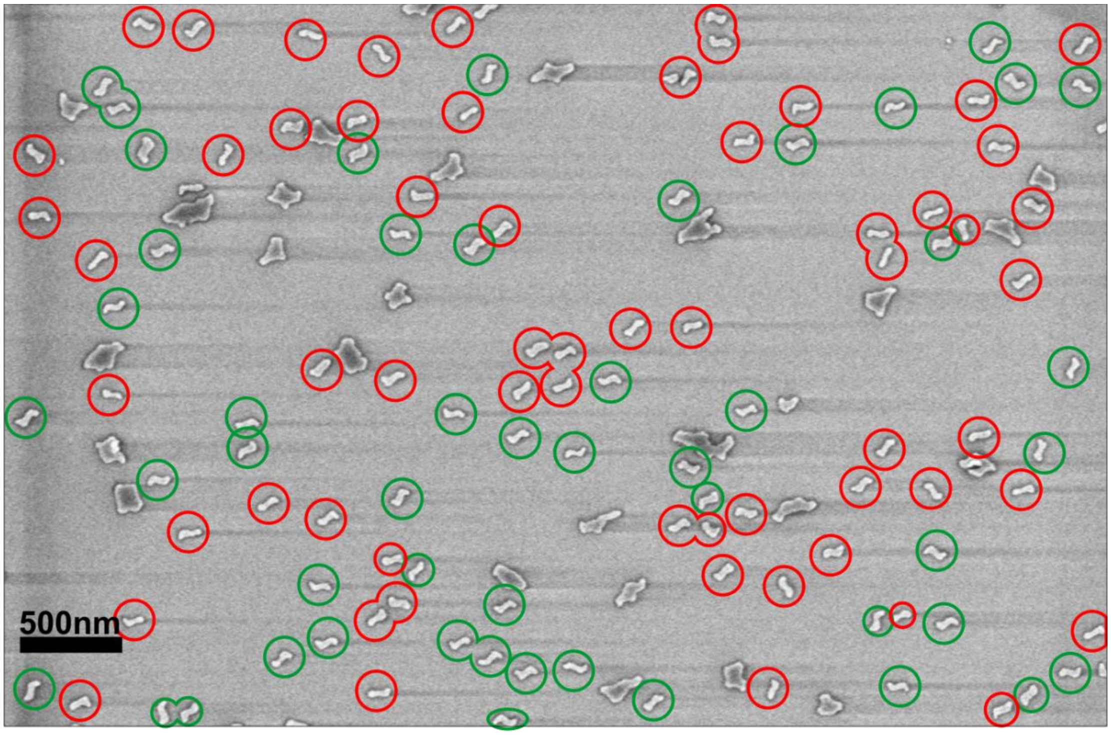

fig. SB4. SEM image of the metallic CDLs (CDL-origamis without staple extensions). The sample are made from 18 nm gold with 2 nm chromium layer. Correctly and malformed CDLs are marked with green and red circles respectively.

To evaluate the metallization yield of the CDL with and without the staple extensions (Figs. S15 and S16) in respect to the possible chiral response in the visible or near infrared spectrum, we counted the correctly formed metallic structures on the sapphire surface. This choice mainly excludes larger aggregates and misshaped structures. It was found out that the CDL with staple extensions had 99 % selectivity towards the intended S-conformation (see Figs. S17 and S18), whereas the deposition of CDLs without the staple extensions yielded a distribution of 51 % of S-shaped and 49 % of Z-shaped CDLs.

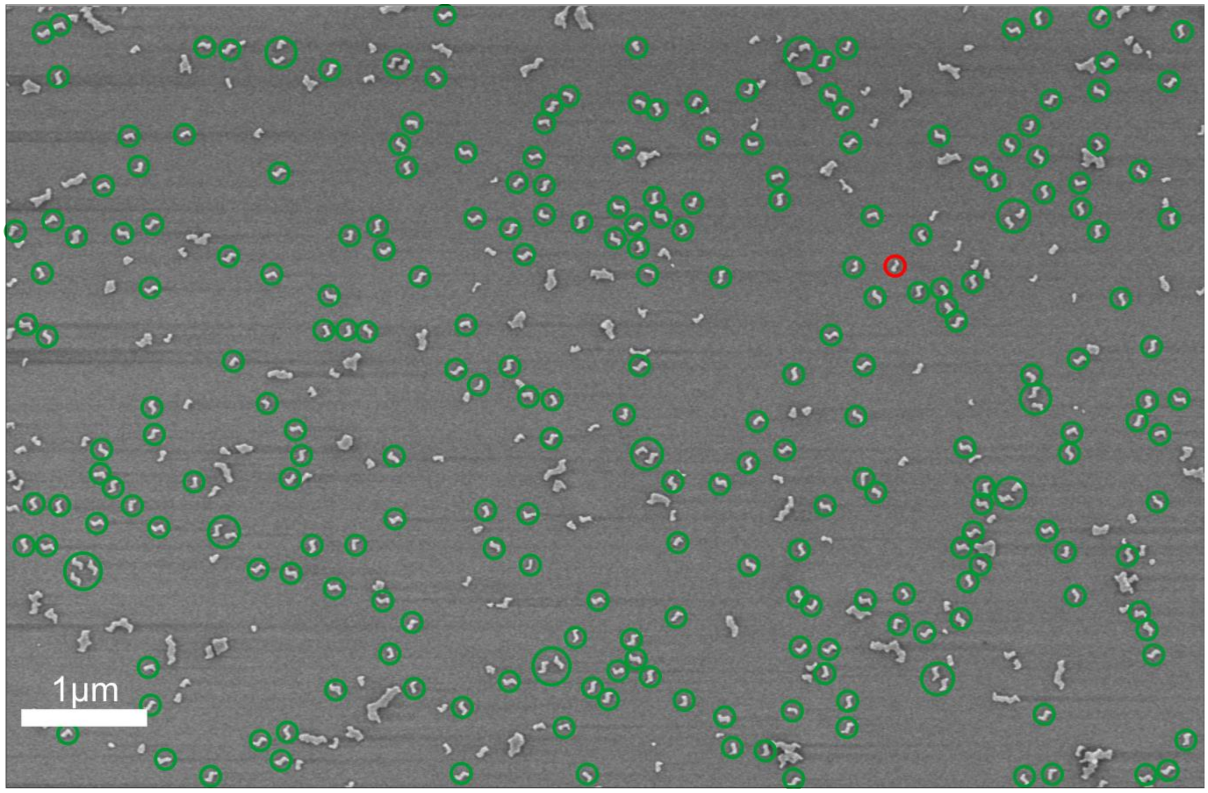

fig. SB5. SEM image of the metallic CDLs (CDL-origamis equipped with staple extensions). The sample are made from 18 nm gold with 2 nm chromium layer. S- and Z-conformations are marked with green and red circles respectively.

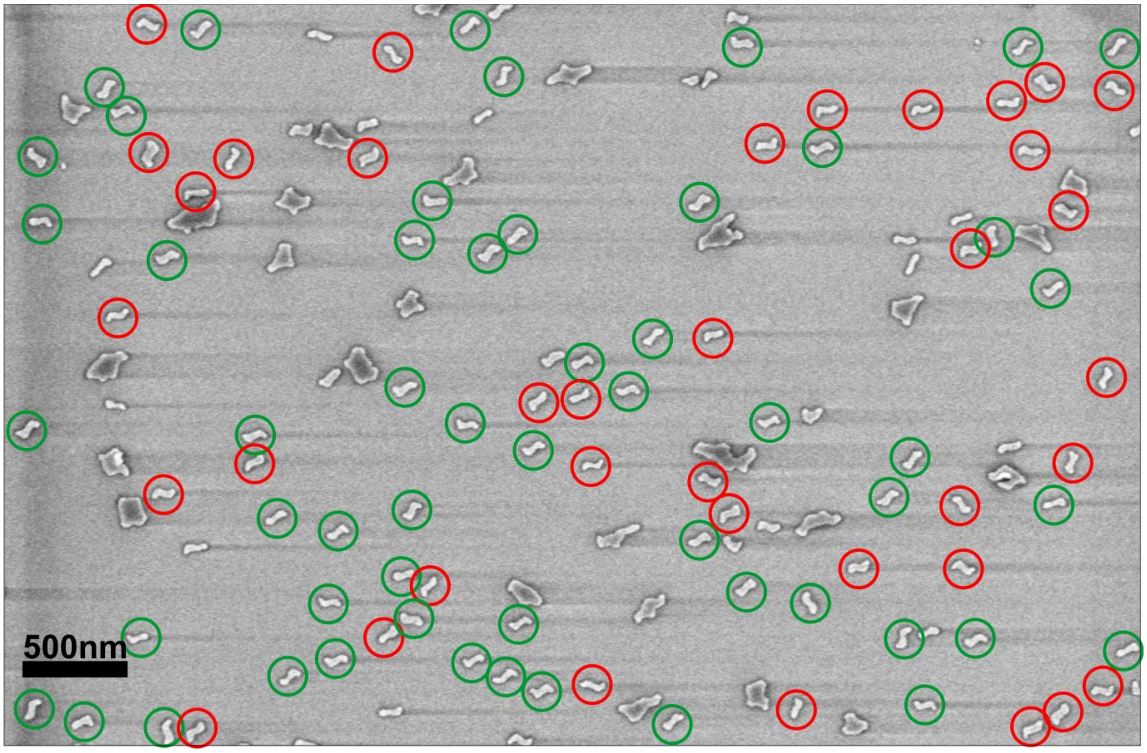

fig. SB6. SEM image of the metallic CDLs (CDL-origamis without staple extensions). The sample are made from 18 nm gold with 2 nm chromium layer. S- and Z-conformations are marked with green and red circles respectively**.**
